# CD73/adenosine dynamics in treatment-induced pneumonitis: balancing efficacy with risks of adverse events in combined radio-immunotherapies

**DOI:** 10.3389/fcell.2024.1471072

**Published:** 2025-01-13

**Authors:** Lena Gockeln, Florian Wirsdörfer, Verena Jendrossek

**Affiliations:** Institute of Cell Biology (Cancer Research), University of Duisburg-Essen, Essen, Germany

**Keywords:** CD73, adenosine, radiotherapy, radio-immunotherapy, immune-related adverse event (irAE), immune checkpoint inhibition, radiation pneumonitis (RP), lung cancer

## Abstract

Consolidation with PD-1/PD-L1-based immune checkpoint blockade after concurrent platinum-based chemo-radiotherapy has become the new standard of care for advanced stage III unresectable non-small cell lung cancer (NSCLC) patients. In order to further improve therapy outcomes, innovative combinatorial treatment strategies aim to target additional immunosuppressive barriers in the tumor microenvironment such as the CD73/adenosine pathway. CD73 and adenosine are known as crucial endogenous regulators of lung homeostasis and inflammation, but also contribute to an immunosuppressive tumor microenvironment. Furthermore, the CD73/adenosine pathway can also limit the immune-activating effects of cytotoxic therapies by degrading the pro-inflammatory danger molecule ATP, which is released into the tumor microenvironment and normal lung tissue upon therapy-induced cell damage. Thus, while targeting CD73 may enhance the efficacy of radio-immunotherapies in cancer treatment by mitigating tumor immune escape and improving immune-mediated tumor killing, it also raises concerns about increased immune-related adverse events (irAEs) in the normal tissue. In fact, combined radio-immunotherapies bear an increased risk of irAEs in the lungs, and additional pharmacologic inhibition of CD73 may further enhance the risk of overwhelming or overlapping pulmonary toxicity and thereby limit therapy outcome. This review explores how therapeutic interventions targeting CD73/adenosine dynamics could enhance radiation-induced immune activation in combined radio-immunotherapies, whilst potentially driving irAEs in the lung. We specifically investigate the interactions between radiotherapy and the CD73/adenosine pathway in radiation pneumonitis. Additionally, we compare the incidence of (radiation) pneumonitis reported in relevant trials to determine if there is an increased risk of irAEs in the clinical setting. By understanding these dynamics, we aim to inform future strategies for optimizing radio-immunotherapy regimens, ensuring effective cancer control while preserving pulmonary integrity and patient quality of life.

## 1 Introduction

Immune checkpoint inhibitors (ICIs) have revolutionized the treatment of solid tumors and are already integrated in the standard treatment of advanced non-small cell lung cancer (NSCLC) (Antonia et al., 2017). At present, their implementation into the clinic is most advanced for ICIs targeting CTLA-4, PD-1 and the PD-1 ligand 1 (PD-L1) (He and Xu, 2020; Shiravand et al., 2022). To increase their therapeutic effectiveness, ICIs are frequently combined with conventional chemotherapy and/or radiotherapy (RT) (Yap et al., 2021; Vafaei et al., 2022; Walsh et al., 2023). These treatments exert direct cytotoxic effects on tumor cells, but also induce inflammatory responses and even prime antigen-specific immune responses, particularly when combined with ICIs. However, since there is still a significant proportion of non-responders and also resistance development, novel therapeutics targeting other immune checkpoints or immunosuppressive pathways in the tumor microenvironment are constantly being developed and tested in clinical trials (Zhou and Yang, 2023). One of these targets is the ecto-5′-nucleotidase CD73, a membrane-bound enzyme of the purinergic signaling pathway. Pro-inflammatory extracellular adenosine triphosphate (ATP) is degraded via the ecto-apyrase CD39 into adenosine monophosphate (AMP). CD73 further converts extracellular AMP into adenosine, which is known to balance inflammatory processes in normal tissues and to promote immune escape in the tumor microenvironment (Yang et al., 2021; Han et al., 2022; Bach et al., 2023; Kaur and Dora, 2023; Kowash and Akbay, 2023; Stagg et al., 2023).

Besides their beneficial use in reactivating an anti-tumoral immune response, ICIs are also associated with a new class of alarming toxicities termed immune-related adverse events (irAEs), which include ICI pneumonitis (Naidoo et al., 2020; Nishimura et al., 2022). This is of clinical importance, as thoracic radiotherapy can trigger radiation pneumonitis in sensitive patients. Although data from clinical trials to date suggest that the combination of chemo-radiotherapy and ICIs only slightly increases toxicity to the irradiated normal tissue, potential interactions that drive pulmonary adverse events in individual patients are not well understood (Antonia et al., 2017). Herein, it is increasingly acknowledged that heterogeneity in the sensitivity of individual patients to inflammation-associated adverse effects induced by radiotherapy, immunotherapy or their combination(s) depends not only on drug concentration, RT dose and quality, and treatment schedule, but is additionally influenced by patient-specific molecular, health, and environmental factors (Chen et al., 2021; Yamaguchi et al., 2022).

The results of the phase III *PACIFIC* trial (NCT02125461) established the current standard of care for patients with unresectable stage III NSCLC: platinum-based concurrent chemoradiotherapy (cCRT) followed by consolidation treatment with the anti-PD-L1 monoclonal antibody Durvalumab for 1 year (Antonia et al., 2017; 2018). Building on these findings, newer trials (i.e., *COAST* and *PACIFIC-9*) are exploring potential improvements by combining cCRT with dual immune checkpoint inhibition targeting both, PD-L1 and CD73. These adaptations could enhance the anti-tumor immune response and address immune evasion mechanisms that might limit the efficacy of PD-L1 monotherapy (Herbst et al., 2022; Bendell et al., 2023). Yet, increasing the complexity of combined treatment concepts by adding CD73-targeted immunotherapy will add to the disturbance of pulmonary immune homeostasis and the potential occurrence of overlapping irAEs in NSCLC treatment and also further complicate adequate risk assessment, diagnosis, and therapy of pneumonitis.

In this review, we assess how therapeutic intervention in CD73/adenosine dynamics could synergize with radiation-induced immune activation in combined radio-immunotherapy concepts to drive irAEs in the lung. Here, we focus on interactions between radiotherapy and CD73/adenosine-targeting strategies in subacute pneumonitis that need to be distinguished from potential overlapping chronic adverse effects (e.g., pulmonary fibrosis), which we have already reviewed elsewhere (De Leve et al., 2019a; De Leve et al., 2019b). In addition, we compare the incidence of (radiation) pneumonitis reported for the *PACIFIC* regimen, with and without additive targeting of CD73, to assess how this alters the risk for irAEs in the clinical setting. Finally, we draw clinically relevant conclusions, stressing the necessity of an individualized irAE risk profile assessment in the planning of combined radio-immunotherapies targeting CD73 and further research on this topic.

## 2 Immune-regulatory functions of CD73 and the purinergic signaling complex

Purine nucleotides and nucleosides are crucial components of intracellular metabolic processes and nucleic acid biomolecules. In addition, especially adenine-based purines also mediate extracellular purinergic signaling, an evolutionally conserved pathway with multifaceted roles in homeostasis and disease, including the regulation of immune responses (De Leve et al., 2019a). In this section, we introduce the purinergic signaling complex, describe how CD73 mediates the resolution of immune responses and how its immune-regulatory function is harnessed in cancer treatment.

### 2.1 The purinergic signaling pathway

**FIGURE 1:**
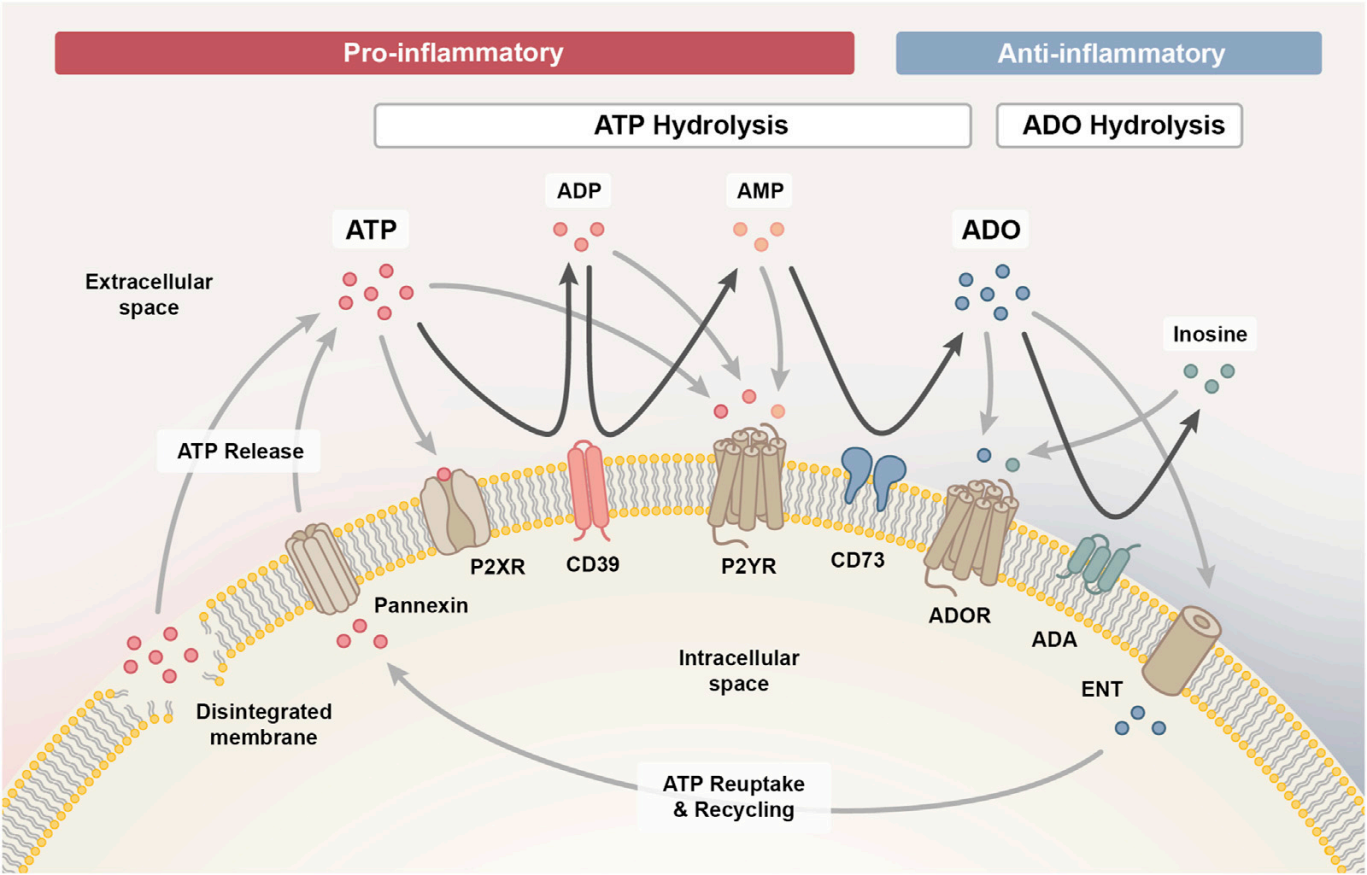
The purinergic signalling system. The purinergic signalling system comprises channels and transporters for purine release and re-uptake, membrane-bound enzymes for extracellular purine hydrolysis and respective P1, P2X and P2Y signalling receptors, which collectively coordinate the initiation and resolution of immune responses. Upon cellular damage, adenosine triphosphate (ATP) enters the extracellular space via pannexin channels or uncontrolled leakage. ATP signalling via P2X and P2Y receptors exerts predominantly pro-inflammatory effects, which facilitates the initiation of an inflammatory response required for the clearance of damaged cells. As the pro-inflammatory ATP pool is hydrolysed into adenosine diphosphate (ADP) and adenosine monophosphate (AMP) signalling intermediates via ectonucleotidase CD39 (ectonucleoside triphosphate diphosphohydrolase 1, ENTPD1) and further into adenosine (ADO) via CD73 (ecto-5′-nucleotidase, NT5E), the immune response transitions from the inflammatory towards the resolution and repair phase. AMP can also be generated through the non-canonical ENPP1 (ectonucleotide pyrophosphate phosphodiesterase 1) pathway (not shown), complementing the canonical CD39 pathway. Adenosine signalling via the P1 purinergic receptors A1, A2A, A2B and A3 primarily exerts anti-inflammatory effects on immune cells and is either reduced when adenosine is hydrolysed to inosine by membrane-bound adenosine deaminase (ADA) or re-entering the cell via concentrative or equilibrative nucleoside transporters (CNTs/ENTs) to be recycled to ATP. In addition, inosine can likewise interact with the A2A and A3 receptor to mediate anti-inflammatory responses.

The entirety of molecules involved in the purinergic signaling system comprises various channels and transporters for purine release and re-uptake, membrane-bound enzymes for extracellular purine hydrolysis, and respective signaling receptors for purine nucleosides and nucleotides, which collectively enable a high degree of signaling complexity (Figure 1) (Giuliani et al., 2021).

Upon cell stress or damage, ATP can enter the extracellular space, either through uncontrolled leakage from disintegrated cell membranes or through controlled release via various means, such as pannexin 1 channels (Cekic and Linden, 2016). Extracellularly accumulating ATP functions as a danger signal and mediates paracrine or autocrine signaling via binding to ionotropic P2X or metabotropic P2Y receptors on the cell surface (De Leve et al., 2019b). P2X receptors are ligand-gated cation channels with seven different subtypes that function as homo- or heterotrimeric complexes specifically activated by ATP, while the eight recognized P2Y receptors are G protein-coupled receptors with differential specificity and affinity for ATP and ADP, as well as the respective uracil-based analogues, UTP and UDP (Kaur and Dora, 2023).

Signaling via P2X and P2Y receptors is terminated when extracellular ATP gets hydrolyzed into adenosine diphosphate (ADP), adenosine monophosphate (AMP) and adenosine signaling intermediates in a stepwise process mediated by membrane-bound ectonucleotidases. Initially, CD39 (ectonucleoside triphosphate diphosphohydrolase 1, ENTPD1) converts ATP into ADP and further into AMP (canonical pathway) (De Leve et al., 2019b). It is important to note that AMP can also be derived independent of CD39 via the CD38/ENPP1 axis (non-canonical pathway) (Ferretti et al., 2019). ENPP1/CD203a/PC-1 (ectonucleotide pyrophosphate phosphodiesterase 1) converts various substrates, including NAD^+^ (nicotinamide adenine dinucleotide), adenosine diphosphate ribose (ADPR), cGAMP and ATP, further contributing to the accumulation of AMP in the extracellular space (Ruiz-Fernandez de Cordoba et al., 2023). CD73 (ecto-5′-nucleotidase, NT5E) is the ectoenzyme ultimately hydrolyzing the remaining 5′ phosphate group from AMP to generate adenosine as the final product. Albeit, adenosine may also be directly released into the extracellular space from stressed or damaged cells (De Leve et al., 2019b).

Extracellular adenosine activates P1 purinergic receptors, a group of G protein-coupled receptors comprising four subtypes (A1, A2A, A2B and A3), that contrarily regulate adenylyl cyclase (AC). While A2A and A2B receptors are coupled to G_s_ proteins that activate AC to increase the production of intracellular cyclic AMP (cAMP), A1 and A3 receptors are coupled to G_i/o_ proteins that inhibit AC to reduce cAMP levels (Kaur and Dora, 2023). The purinergic receptor subtypes also differ in the signaling pathways they initiate within target cells and in adenosine affinity (A1 > A2A > A3 >> A2B), allowing adenosine to modulate both, routine and stress-induced cellular responses. While physiological adenosine levels are sufficient to activate A1, A2A and A3 receptors, activation of the A2B subtype requires tenfold higher adenosine concentrations, which only prevail in pathological or stressed conditions (De Leve et al., 2019b).

Signaling through P1 receptors recedes when extracellular adenosine is re-entering the cell via concentrative or equilibrative nucleoside transporters (CNTs/ENTs) to be recycled to AMP by intracellular adenosine kinase (ADK) (De Leve et al., 2019b). Alternatively, adenosine can also be deaminated by membrane-bound adenosine deaminase (ADA) to inosine, which likewise functions as an agonist for A2A and A3 receptors, albeit with lower affinity than adenosine and an extracellular-signal regulated kinases (ERK)1/2 downstream signaling bias (Gomez and Sitkovsky, 2003; Welihinda et al., 2016).

### 2.2 Purinergic regulation of immune responses

In addition to its actions on cellular responses such as cell proliferation, differentiation, migration, apoptosis, neurotransmission, secretion, vasodilation and platelet aggregation, purinergic signaling is also crucial for the temporal coordination of immune responses. Most immune cells are equipped with components of the purinergic signaling system and their behavior is accordingly shaped by the cell-specific expression profile of purinergic receptors and ectoenzymes, as well as the local concentrations and ratio of ATP and adenosine. In this context, extracellular ATP primarily exerts pro-inflammatory effects, while adenosine has mostly anti-inflammatory or immunosuppressive properties (Cekic and Linden, 2016; Huang et al., 2021; Kaur and Dora, 2023).

#### 2.2.1 Acute phase

In the acute phase after tissue injury, the rapid accumulation of ATP released from stressed and damaged cells into the extracellular space facilitates the recruitment and activation of various immune cell types to initiate a robust immune response (Cekic and Linden, 2016). In particular, extracellular ATP functions as a chemoattractant for phagocytes (monocytes, macrophages, DCs and neutrophils) and stimulates immune cell activation. Signaling through P2X and P2Y receptors, which are highly expressed on monocytes and macrophages, stimulates activation of the inflammasome. Further, extracellular ATP promotes effector T cell receptor (TCR) signaling, while regulatory T cell (T_reg_) functionality and survival are diminished (Cekic and Linden, 2016).

#### 2.2.2 Subacute phase

In the subacute phase, the enhanced activity of CD39 and CD73 ectonucleotidases increases the local concentration of adenosine in order to limit the inflammatory response. Activated immune cells also upregulate the G_s_-coupled P1 receptor subtypes A2A and A2B, which increases their sensitivity to extracellular adenosine. A2A receptors are expressed on the majority of immune cells (T cells, B cells, monocytes, macrophages, dendritic cells (DCs), natural killer (NK) cells, mast cells, eosinophils and platelets). In contrast, A2B receptors, which require higher adenosine concentrations for their activation, are primarily expressed on the surface of macrophages and DCs, and to a lower extent also on lymphocytes and platelets (Cekic and Linden, 2016). Adenosine signaling through A2A and A2B receptors activates AC to increase intracellular cAMP levels, which stimulate the production of anti-inflammatory cytokines (e.g., IL-10), while pro-inflammatory cytokines [e.g., tumor necrosis factor alpha (TNFα) or IL-1β] are downregulated (Wang et al., 2024). Further, adenosine inhibits phagocyte chemotaxis and activation, as well as antigen-presentation. Adenosine signaling limits effector T cell responses directly by suppressing cytokine secretion, T cell infiltration and *de novo* activation, but also by promoting exhaustion of already activated effector T cells (Wang et al., 2024). While adenosine restrains pro-inflammatory immune cell activities, it likewise fosters the expansion and polarization of tolerance-associated immune cell types, such as T_regs_, M2 macrophages and myeloid-derived suppressor cells (MDSCs) (De Leve et al., 2019a). Besides its catalytic activity, CD73 on endothelial cells can also function physically as an adhesion receptor and is thus involved in orchestrating leukocyte trafficking in response to chemotactic stimuli and regulating vascular permeability (Eltzschig et al., 2004; Thompson et al., 2004; Salmi and Jalkanen, 2005; Linden, 2006; Eckle et al., 2007; Ålgars et al., 2011). Eventually, deamination of the accumulating adenosine pool by ADA generates inosine, which has a longer half-life than adenosine and can amplify and prolong its anti-inflammatory effects (Welihinda et al., 2016).

#### 2.2.3 Chronic phase

As the inflammatory response progresses further towards the chronic phase, the ATP/adenosine ratio continues to decline and mediates the initiation of wound healing processes. Yet, a prolonged increase in extracellular adenosine levels and hence persistent A2B receptor signaling at this stage can escalate to fibrotic tissue remodeling, mainly via upregulated secretion of IL-6 and vascular endothelial growth factor (VEGF), alternatively polarized macrophages and T_h_17 cells (Wirsdörfer et al., 2013; 2019; De Leve et al., 2017).

### 2.3 Pharmacologic targeting of CD73 to boost anti-tumor immunity

CD73 and other components of the purinergic system are aberrantly expressed in numerous cancers and are considered as important regulators of an adverse tumor microenvironment. For example, chronically inflamed and hypoxic tumor microenvironments are associated with upregulated CD73 expression as well as deregulated levels of ATP and adenosine (Vaupel and Mayer, 2016; Chambers and Matosevic, 2019). In this regard, CD39 and CD73 play key roles in generating adenosine-enriched immunosuppressed and pro-angiogenic environments that support cancer development and malignant tumor (cell) behavior (Antonioli et al., 2016; Pacheco and Schenk, 2021). As adenosine-mediated immunosuppression constitutes one of many mechanisms utilized by tumors to escape from neoantigen-induced anti-tumor immune responses, overexpression of CD39, CD73 and A2A receptors in the tumor microenvironment further correlates with poor survival and therapy response. High intrinsic CD73/adenosine signaling in the tumor microenvironment may also restrain radiotherapy-induced antitumor immune responses and thereby limit the efficacy of combined radio-immunotherapies (Vaupel and Multhoff, 2016; Wennerberg et al., 2017). Consequently, adenosine receptor antagonism or pharmacologic inhibition of ectonucleotidase activity to restrain ATP hydrolysis and downstream generation of adenosine are promising strategies to improve the treatment of various solid tumors, including NSCLC. Furthermore, these factors are also used as prognostic biomarkers for various tumors (Wang et al., 2024).

CD73 is highly expressed in NSCLC and its expression correlates with and is regulated by common oncogenic drivers like Kirsten rat sarcoma virus (KRAS), epidermal growth factor receptor (EGFR), mitogen-activated extracellular signal-regulated kinase (MEK) or anaplastic lymphoma kinase (ALK) (Han et al., 2022; Saigí et al., 2023). Investigations on the pro-tumorigenic role of CD73 demonstrate that CD73 impacts various cancer hallmarks, such as tumor cell cycle progression, invasiveness, epithelial-mesenchymal transition (EMT), angiogenesis, migration and metastasis (Kowash and Akbay, 2023). In its function as an immune checkpoint, CD73 generates adenosine that establishes an immunosuppressive tumor microenvironment, primarily by interfering with effector T cell, DC and NK cell expansion and/or functionality. At the same time, CD73-derived adenosine fosters M2-polarized macrophages, T_regs_ and MDSCs (Kowash and Akbay, 2023; Saigí et al., 2023). While preclinical models revealed favorable anti-tumor effects of pharmacologic CD73 inhibition, treatment strategies combining CD73 blockade with standard chemo-radiotherapy or other immune checkpoint inhibitors warrant further investigation.

Various CD73-targeting pharmaceutics have proceeded to clinical testing, including the anti-CD73 monoclonal antibody (mAB) Oleclumab (MEDI9447), which selectively binds CD73 to inhibit its catalytic activity by steric blocking and dimer crosslinking and also increases CD73 internalization. The first in-human study of Oleclumab was a phase I clinical trial (NCT02503774) running from 2015 to 2023 that aimed to evaluate its efficacy and safety alone or in combination with Durvalumab (anti-PD-L1 mAB) in patients with advanced solid tumors (Bendell et al., 2023). At present, targeting of CD73 with Oleclumab is considered for combined radio-chemo-immunotherapy concepts to improve the survival of stage III unresectable NSCLC patients (*COAST* and *PACIFIC-9* clinical trials) (Herbst et al., 2022; Barlesi et al., 2024).

## 3 Crossed immunomodulatory paths: How CD73-directed immunotherapy could exacerbate radiation pneumonitis

Pharmacologic targeting of immune checkpoints like CD73 has substantial therapeutic potential in cancer treatment, but in addition to a (re-)activation of the anti-tumoral immune response, ICIs can also trigger a specific class of toxicities, irAEs, which include ICI pneumonitis (Guberina et al., 2023). It is evident that ICIs are particularly effective when combined with conventional immune-activating therapies, such as radiotherapy. The therapeutic effect of radiotherapy is based on the complex local damage that ionizing radiation exerts to cellular macromolecules, particularly to the DNA, which commonly results in cell growth arrest or death. Yet, the discovery that ionizing radiation has also systemic effects and can boost anti-tumor immunity, introduced a paradigm shift and provides rationales for combining radio- and immunotherapies to achieve a synergistic treatment effect: These effects include, on the one hand, activation of local and systemic immune response mechanisms via classic damage-signaling cascades and immunogenic cell death [e.g., recognition of nuclear and mitochondrial DNA fragments via cyclic GMP-AMP synthase (cGAS)/stimulator of interferon genes (STING), recognition of damage-associated molecular patterns (DAMPs) by, e.g., TOLL-like receptors (TLRs), immune-mediators from the senescence-associated secretory phenotype (SASPs) and inflammasome activation] and, on the other hand, enhancement of tumor antigen presentation (e.g., enhancing the exposure of immunogenic mutations frequently contained in DNA damage repair genes and increasing the release of tumor antigens upon cell death induction) (Wirsdörfer and Jendrossek, 2017; Lhuillier et al., 2019; Wirsdörfer et al., 2019; Guberina et al., 2023). Importantly, radiotherapy also mediates an increased cell surface expression of various immune checkpoints, including CD73, which represents an additional rationale for combined radio-immunotherapy (Mohamed et al., 2023).

Similar to ICIs, radiotherapy as such can thus also initiate irAEs. This observation is of particular clinical importance for thoracic radiotherapy, as exposure of the intrinsically radiosensitive normal lung tissue to ionizing radiation can trigger radiation pneumonitis, a late-occurring and potentially life-threatening immune-related complication of radiotherapy. The diagnosis of radiation pneumonitis is based on an increased density of the lung parenchyma in CT monitoring in conjunction with a recent history of thorax radiotherapy and the exclusion of other causes. Classic pneumonitis symptoms like cough, dyspnea, low-grade fever and chest pain commonly occur within 3–12 weeks after radiation exposure, but are not necessarily present. As these symptoms are non-specific and occurring with a delay, the clinical distinction between radiation pneumonitis and similarly presenting lung diseases such as ICI pneumonitis or pneumonia is challenging and contributes to the fact that radiation pneumonitis may not be recognized immediately in follow-up care (Bledsoe et al., 2017; Giuranno et al., 2019; Rahi et al., 2021; Guberina et al., 2023).

As the occurrence of radiation pneumonitis depends on patient-, tumor- and treatment-related factors, a wide range of clinical incidence rates for radiation pneumonitis is reported in the literature. For patients with stage III unresectable NSCLC treated with chemo-radiotherapy, the incidence of grade 3–5 radiation pneumonitis reported in a recent meta-analysis ranged from 3.62% to 7.85%, yet considerably higher incidence rates have been observed in vulnerable patient cohorts with underlying pulmonary dysfunction (Zhou Y. et al., 2020; Kuang et al., 2022). Although severe radiation pneumonitis is a rare complication of thoracic radiotherapy, mainly due to technical and physical innovations that have helped to enhance physical accuracy of dose delivery, it is nonetheless devastating for the prognosis of affected patients as only symptomatic treatment options are available. Thus, radiation pneumonitis remains a major dose-limiting factor.

With the addition of immuno-therapeutics to chemo-radiotherapy regimens, a novel, potentially overlapping risk factor for the development of pneumonitis has been introduced, which necessitates the re-evaluation of current risk assessments for each combinatorial treatment regimen. However, this type of investigation is still a largely neglected field of research in radiobiology.

Consolidation therapy with PD-1 and PD-L1 immune checkpoint inhibition has already been shown to increase the risk for radiation pneumonitis, as reviewed elsewhere (Chen et al., 2023). In the following paragraphs, we delineate the mechanisms driving radiation pneumonitis and elaborate how CD73-directed immunotherapy might interfere with disease pathogenesis or severity based on shared immunomodulatory pathways.

### 3.1 Radiation-induced immune response of the normal lung tissue

Radiation-induced lung injury (RILI), which comprises subacute radiation pneumonitis (RP) and chronic radiation-induced lung fibrosis (RILF), is initiated by off-target radiation to non-immune and immune cells of the healthy lung tissue, causing an exaggerated immune response. The initial damage to cellular macromolecules, in particular the cellular DNA, can be induced directly, but up to 60% is induced indirectly via generation of reactive oxygen and nitrogen species (ROS/RNS), which can be even more detrimental, as they amplify, persist and spread to cells outside of the radiation field (Barcellos-Hoff et al., 2005; Azzam et al., 2012; Schipler and Iliakis, 2013; Giuranno et al., 2019). Based on cell-specific properties, the exposure to ionizing radiation leads to a cell type-specific damage load and stress response, with higher damage-loads usually triggering stronger immunogenic processes. At later stages, chronically alternating inflammation and tissue repair cycles without attaining actual resolution may cause progression to fibrotic tissue remodeling of the lungs, as nicely summarized by Jarzebska et al. (2021). The majority of the current knowledge on the mechanisms driving RILI has been gained in preclinical investigations in rodents and is described in the following paragraphs.

#### 3.1.1 Acute phase

The cellular response to the initial radiation damage, ranging from transient cell cycle arrest and recovery, over senescence and immunologically silent forms of cell death (e.g., apoptosis), to more immunogenic forms of cell death, is dependent on intrinsic cell properties (e.g., intrinsic radiosensitivity, DNA repair capacity and turnover kinetics), and decisive for the impact on the immune system (Denekamp and Rojas, 1989; Rosen et al., 1999; Golden et al., 2014; Bekker et al., 2022). Immune cells show a cell-type specific spectrum of radiosensitivity, but are in general considered to be highly susceptible to ionizing radiation. Hence, upon radiation exposure of the lungs, an acute but transient loss of various immune cells in the local tissue and also in the peripheral blood can be observed (Belka et al., 1999). This acute immune cell depletion is followed by a time-dependent immune cell recruitment, which establishes a sterile pro-inflammatory lung environment that facilitates the removal of injured cells to enable tissue restoration and the return to homeostasis (Belka et al., 1999; Heylmann et al., 2014; Wirsdörfer et al., 2014; Cytlak et al., 2022).

Radiation-induced immune activation is initially driven by classic tissue damage response pathways (mainly DAMP sensing, cGAS/STING-mediated DNA damage signaling and inflammasome activation) (Wirsdörfer et al., 2019): Shortly after damage induction (radiation), a spectrum of DAMPs is released from damaged cells into the extracellular space [e.g., ATP, high mobility group box chromosomal protein B1 (HMGB1), heat shock protein 70 (HSP70), uric acid, fragmented extracellular matrix molecules like low molecular weight hyaluronan (HA) and altered nucleic acids] or expressed on the cell surface [e.g., calreticulin (CRT)] (Land, 2015; Wirsdörfer and Jendrossek, 2017; Murao et al., 2021; Boerma et al., 2022). These damage signals are recognized by pattern recognition receptors (PRRs), such as TLR2 and TLR4, expressed on the surface of most non-immune cells [e.g., endothelial cells, alveolar epithelial cells (AECII) and fibroblasts] and also immune cells (e.g., monocytes, alveolar macrophages, neutrophils, DCs, NK cells and lymphocytes) (Jarzebska et al., 2021; Boerma et al., 2022). Damage sensing and PRR signaling initiate the sequential release of pro-inflammatory mediators, thereby fostering the time-dependent recruitment and activation of various innate and adaptive immune cells (Wirsdörfer et al., 2019).

Recruited immune cells respond with the production of further pro-inflammatory mediators. The cascade of inflammatory cytokines in the irradiated lung tissue can be further reinforced by activation of intracellular inflammasome protein complexes, for instance via binding of extracellular ATP to P2X7R purinoceptors. NOD-, LRR- and pyrine domain-containing protein 3 (NLRP3) inflammasome activation leads to the auto-catalytic cleavage of inactive pro-caspase-1 that in turn cleaves inactive precursors of the IL-1 family (IL-1β and IL-18), which bind to IL-1R-1, stimulating the production of further cytokines, like TNFα, IL-6 and also pro-IL-1β, that recruit and activate additional immune cells, establishing a robust sterile inflammation (Wirsdörfer and Jendrossek, 2017; Jarzebska et al., 2021). Inflammatory cytokines (TNFα, IL-6, type-I interferons) might also be induced upon sensing of altered cytosolic DNA species by cGAS, an intracellular PRR, which leads to phosphorylation of STING and the activation of nuclear factor kappa B (NF-κB) and interferon regulatory factor 3 (IRF3) (Yang et al., 2023).

In the course of the inflammatory response, dynamic immune cell composition changes and the transition from innate to adaptive immunity can be observed. As such, neutrophils and NK cells are attracted early by ROS and RNS species to initiate innate phagocytic and cytotoxic responses (Cytlak et al., 2022). Later, DAMPs function as a “find-me” signal, which is sensed by inflammatory monocytes and macrophages via their PRR to direct them to the site of injury and to encourage the removal of damaged cells. Further, the translocation of CRT to the cell surface of damaged cells undergoing immunogenic cell death in conjunction with HMGB1-TLR4 and ATP-P2XR7 damage signaling enables the maturation of DCs to prime adaptive immune cell responses (Elliott et al., 2009; Golden et al., 2014; Jarzebska et al., 2021; Cytlak et al., 2022).

#### 3.1.2 Subacute phase

In the subacute phase, the sterile inflammation in the lungs is reinforced via several mechanisms: As the inflammatory phase is marked by T_h_1 T cells, large amounts of pro-inflammatory cytokines like interferon gamma (IFNγ) are secreted, which, among other processes, promote the M1 polarization of macrophages that express inducible nitric oxide synthase (iNOS). Other pro-inflammatory mediators like TNFα and IL-1β activate iNOS to catalyze the production of large nitric oxide (NO) amounts, which cause secondary macromolecular damage that subsequently promotes further immune activation and amplification of the inflammatory response (Jarzebska et al., 2021; Yan et al., 2022). Other drivers of immune activation in the subacute phase are resident cells that acquired persistent sublethal radiation damage. As fully differentiated lung cells rarely divide, they are less prone to an immediate cell death upon exposure to ionizing radiation, but rather foster inflammation in the subacute phase when they eventually attempt cell division, which results in mitotic cell death due to previously acquired genome damages. Sublethal radiation damage can also induce permanent cell cycle arrest and the acquisition of a senescent phenotype, particularly in alveolar epithelial and endothelial cell populations. Senescent cells release a characteristic set of soluble mediators, termed the senescence-associated secretory phenotype (SASP), which contributes to the pro-inflammatory micromilieu (Citrin et al., 2013; Wunderlich et al., 2017; Hansel et al., 2020).

Continuous recruitment of immune cells is also promoted by radiation-induced damage to the alveolar endothelium via upregulation of adhesion molecules, like intracellular adhesion molecule 1 (ICAM-1) or vascular cell adhesion molecule 1 (VCAM-1), as well as structural changes to the endothelial glycocalyx, which facilitates the interaction with circulating leukocytes (Klein et al., 2016; Käsmann et al., 2020; Cytlak et al., 2022). The progressive loss of endothelial cells further results in vascular leakage and over time in oedema formation and chronic tissue hypoxia (Venkatesulu et al., 2018). Likewise, the loss of AECI and their reconstitution from AECII cells causes diminished production of alveolar surfactant, resulting in the loss of barrier function and increased alveolar surface tension in the long term. In addition, the dysfunction of AECII cells can cause deregulation of alveolar macrophages, which reside in close proximity to AECII cells for mutual regulation under homeostatic conditions (Bissonnette et al., 2020).

**FIGURE 2:**
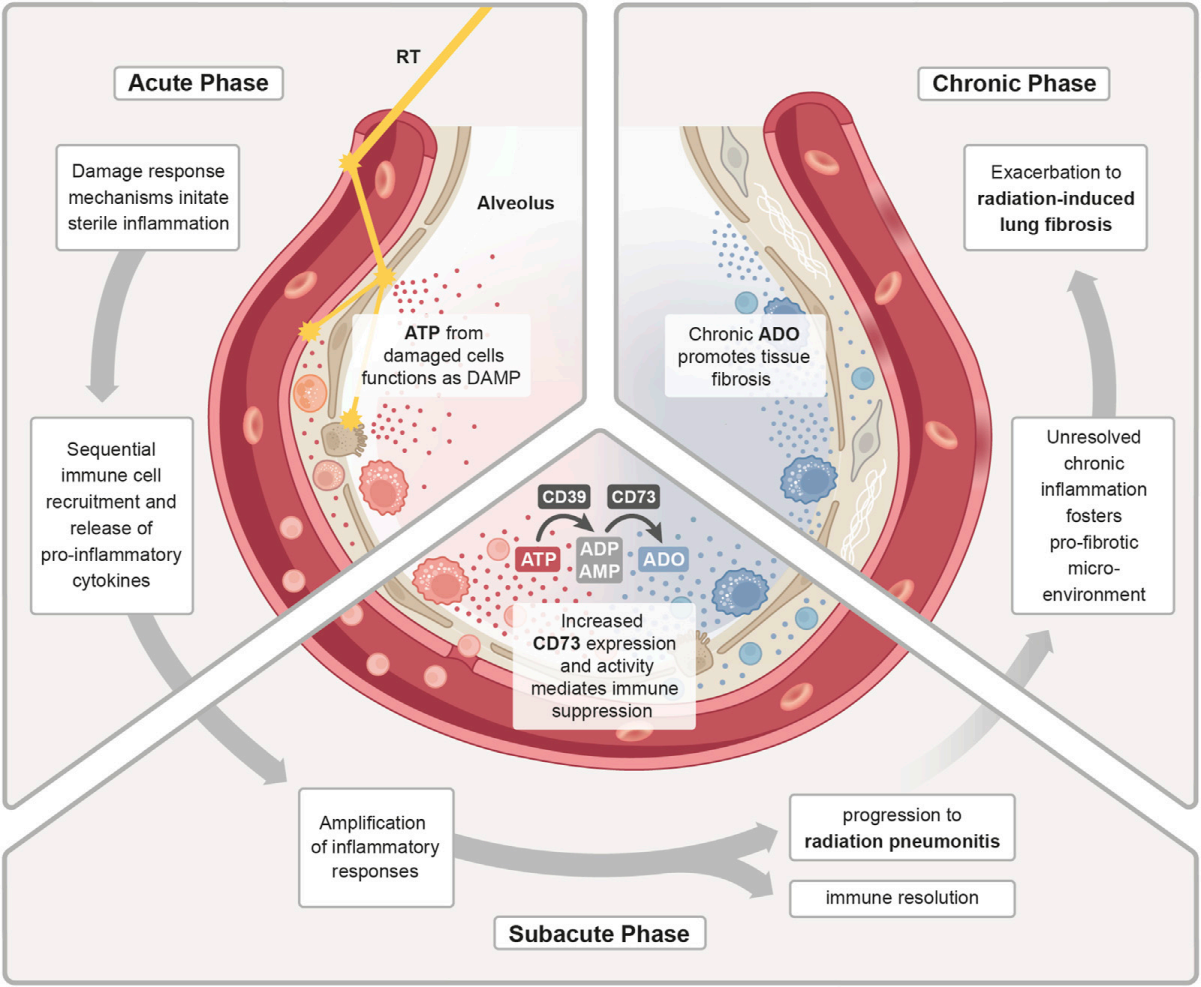
ATP/adenosine dynamics in radiation pneumonitis. Treatment with radiotherapy (RT) induces a damage response in the malignant and normal lung tissue. In the **acute phase**, release of adenosine triphosphate (ATP) from damaged cells functions as a DAMP to initiate a sterile inflammation. Subsequent immune cell recruitment and release of cytokines further fuels inflammation. During the **sub-acute phase**, the inflammatory response is further amplified and as a counteracting mechanism increased expression and activity of CD73 enhances the conversion of pro-inflammatory ATP into anti-inflammatory adenosine (ADO). The homeostatic regulation of inflammatory responses is needed to promote immune resolution and tissue regeneration. Uncontrolled, excessive pro-inflammatory responses can lead to radiation-induced pneumonitis. In the **chronic phase**, continuous cycles of secondary tissue damage and persistence of inflammatory drivers further changes the lung environment resulting in chronic inflammation and increase in profibrotic mediators (e.g., growth factors, TGFβ, hypoxia). Chronic ADO promotes this pro-fibrotic micromilieu which fosters fibroblast recruitment/activation and deposition of excessive extracellular matrix molecules resulting in radiation-induced lung fibrosis.

The above-mentioned amplification of the sterile inflammation and the immune-mediated secondary damage during the subacute phase requires counteracting anti-inflammatory mechanisms to keep the immune response under control. Herein, tolerance-associated immune cells like T_regs_ are suspected to counteract inflammatory mechanisms in the irradiated lungs by secreting various anti-inflammatory mediators, [e.g., transforming growth factor beta (TGFβ), IL-10 and IL-13], which suppress pro-inflammatory T cells and stimulate M2 polarization in macrophages (Wirsdörfer et al., 2014; Guo et al., 2020). In this context, also the CD73/adenosine axis can contribute to the resolution of inflammation and return to homeostasis (Figure 2). Early on in the acute radiation response phase of the lungs, extensive amounts of extracellular ATP are released, which functions as a DAMP and induces important pro-inflammatory responses, such as activation of the inflammasome and the subsequent release of inflammatory cytokines (Trautmann, 2009). In the subacute phase, this ATP pool is stepwise converted into adenosine by CD39 and CD73, which terminates P2X and P2Y signaling and enables the activation of P1 receptors. Own work using a murine whole thorax irradiation model revealed that adenosine levels in the lung tissue were significantly increased by week 16 after radiation exposure, and this was accompanied by an increased enzymatic activity of CD73 (Wirsdörfer et al., 2013). Research has demonstrated that adenosine affects a variety of immune cells, primarily mediating immunosuppression through negative feedback loop inhibition of activated immune cells and stimulation of anti-inflammatory subpopulations to stop further tissue damage (Sitkovsky et al., 2004). However, when pro-inflammatory cues continuously dominate and resolution fails, the inflammatory response of the lung tissue can exacerbate into subacute radiation pneumonitis.

#### 3.1.3 Chronic phase

When the ongoing inflammatory radiation response fails to reach true resolution, due to the persistence of inflammatory drivers and inflammation-induced secondary tissue damage, an imbalance of inflammation, counteracting immunosuppressive mechanisms and repair processes can cause fibrotic disease progression. Fibrogenesis is associated with accumulation of chronic adenosine and the pro-fibrotic key mediator TGFβ from multiple cellular sources and a switch from T_h_1/T_h_17 to T_h_2 T cell activities, which can stimulate the M2 polarization in macrophages via secretion of IL-4 and IL-13 (Yan et al., 2022). M2-polarized macrophages can in turn facilitate the activation of latent TGFβ pools. Ultimately, the pro-fibrotic microenvironment fosters an excessive secretion of extracellular matrix molecules (ECM) such as collagens from activated fibroblasts. Additionally, the chronic phase is also marked by an increase in pro-angiogenic factors, like vascular endothelial growth factor (VEGF) or hypoxia inducible factor 1 subunit alpha (HIF1α) (Bledsoe et al., 2017), which promote fibrosis and also stimulate futile angiogenesis.

#### 3.1.4 Context-dependent function of CD73 in the inflammatory and fibrotic phases of radiation-induced lung injury

It is assumed that the role of the CD73/adenosine axis in RILI varies significantly across the acute, sub-acute, and chronic stages, highlighting the context-dependent therapeutic potential of modulating CD73 activity. Both agonistic and antagonistic approaches may therefore offer benefits depending on the phase of disease progression (De Leve et al., 2019a; De Leve et al., 2019b).

In the acute phase of the radiation response, large amounts of extracellular ATP are released from damaged cells, acting as a chemotactic signal that recruits neutrophils, macrophages, and other immune cells to the injury site. These immune cells become activated to clear cellular debris. ATP also activates the NLRP3 inflammasome in macrophages via the P2X7 receptor, triggering the release of pro-inflammatory cytokines, particularly IL-1β and IL-18, which sustain the inflammatory response (Land, 2015; Wirsdörfer and Jendrossek, 2017; Murao et al., 2021; Boerma et al., 2022). Prolonged immune cell recruitment and high ATP levels amplify inflammation and contribute to bystander tissue damage. To counteract this, ATP is converted by CD39/CD73 ectoenzymes into immunosuppressive adenosine during the sub-acute phase, helping to limit excessive inflammation and prevent the development of chronic inflammation (Wirsdörfer et al., 2013). If unresolved, radiation-induced tissue damage, bystander effects, and leaky vasculature promote a persistent state of immune cell infiltration and activation. This chronic inflammation eventually polarizes macrophages from M1 to M2 phenotypes, contributing to a pro-fibrotic environment (Bledsoe et al., 2017; De Leve et al., 2017; Yan et al., 2022). In this context, own data show that loss of CD39 in a murine RILI model potentially fosters ATP-driven chronic inflammation resulting in bystander tissue damage and exacerbated radiation-induced lung fibrosis (Meyer et al., 2020). In the chronic phase, CD39/CD73-mediated ATP conversion likewise promotes pro-fibrotic signaling as sustained extracellular adenosine pools lead to chronic purinergic receptor stimulation leads to a transition from pro-inflammatory signaling to anti-inflammatory and tissue remodeling pathways (Wirsdörfer et al., 2013; De Leve et al., 2019a; De Leve et al., 2019b). Therapeutically, enhancing CD39/CD73-mediated ATP conversion to adenosine may be beneficial in the early phase to control inflammation, whereas antagonizing the purinergic system could help prevent or treat fibrosis in the chronic phase. Various pre-clinical studies have validated therapeutic strategies targeting purinergic signaling in lung injuries. These include modulating purinergic ectoenzymes and targeting purinergic receptors (Reutershan et al., 2007; Folkesson et al., 2012; Hoegl et al., 2015). Despite growing interest in targeting the purinergic system to treat lung injuries, studies specifically examining the role of this pathway in pneumopathies induced by radiation exposure are limited. Our own work with a murine whole-thorax irradiation model demonstrated a progressive increase in CD73 activity and adenosine levels post-irradiation. Notably, CD73-deficient mice showed reduced fibrosis, and pharmacological inhibition of CD73 or adenosine deaminase significantly mitigated fibrosis. This underscores the therapeutic potential of targeting adenosine signaling to alleviate radiation-induced lung damage (Wirsdörfer et al., 2013). Additionally, the adaptive transfer of CD73^+^ mesenchymal stem cells (MSCs) or their secretory products, such as extracellular vesicles (EVs), has shown promise in pre-clinical thorax irradiation models by reducing vascular damage, inflammation, and fibrosis in the lungs (Klein et al., 2016; Klein et al., 2017; Lei et al., 2021; Montay-Gruel et al., 2021; Shao et al., 2021; Hou et al., 2022; Li et al., 2022; Gupta et al., 2024). While CD73 is a required identification marker for MSCs (Dominici et al., 2006), the role of MSC-derived CD73 in RILI remains unclear. Moreover, the immunosuppressive potency of human MSC-EV preparations intended for clinical applications has not been directly attributed to the presence of CD73 (Bauer et al., 2023).

In the context of combined radio-immunotherapies for stage III unresectable NSCLC, CD73 is targeted with intent to sustain radiation-induced immune activation through extracellular ATP. However, the conversion of ATP to adenosine within the tumor microenvironment suppresses DC maturation and inhibits CD8^+^ cytotoxic T cells, thereby weakening antigen presentation and the anti-tumor immune response (Mastelic-Gavillet et al., 2019; Wennerberg et al., 2020; Lin et al., 2023). Furthermore, CD73 activity enhances tumor cell survival, migration, and invasion via adenosine-mediated activation of signaling pathways such as phosphoinositide 3-kinase (PI3K)/protein kinase B (AKT) and mitogen-activated protein kinases (MAPK) (Ma et al., 2019; Zhan et al., 2024). As discussed earlier, the consequences of CD73 modulation in RILI will be highly context-dependent. In current clinical trials (e.g., *COAST*) administration of the CD73-targeting antibody Oleclumab begins 1–42 days after cCRT completion, thus coinciding with the acute and sub-acute post-irradiation phase. However, as immunotherapy is administered as consolidation treatment for up to 1 year, there is also potential for an overlap with the onset of chronic adverse effects, such as fibrosis (Herbst et al., 2022).

In the following section, we explore how therapeutic inhibition of CD73 activity might influence radiation-induced pneumonitis by modulating the immune response. These effects must be differentiated from the potential benefits of CD73 antagonism during chronic tissue remodeling. For comprehensive reviews of the role of CD73/adenosine in radiation-induced tissue fibrosis, refer to De Leve et al. (2019b), De Leve et al. (2019a).

### 3.2 Perspective: how therapeutic targeting of CD73 might interfere with immune regulation in radiation pneumonitis

Therapeutic intervention in the purinergic system could exert multiple immunomodulatory effects in the irradiated lung. In the malignant tissue, where CD73 is already upregulated or induced by radiation, purinergic adenosine production leads to an immunosuppressive adenosine “halo” around the tumor that mediates immune escape as described above and in other recent reviews (Yang et al., 2021; Allard et al., 2023; Bach et al., 2023; Xia et al., 2023). So far, it remains to be determined if CD73-mediated immunomodulation plays a significant role in radiation pneumonitis. In the co-irradiated normal lung tissue, radiation induces a damage response and immune infiltration and subsequent inflammation, potentially exacerbating into radiation pneumonitis. While the acute response to ionizing radiation is dominated by extracellular ATP, the subacute and chronic phase are associated with a decline in the ATP/adenosine ratio (Volmer et al., 2006; Wirsdörfer et al., 2013; Jarzebska et al., 2021). This is a consequence of the inflammation-induced upregulation and increased activity of CD73 on diverse resident lung cells, as well as immune cells in the normal lung tissue, thus contributing to elevated adenosine levels, presumably to dampen overwhelming inflammatory responses and limit radiation pneumonitis as shown in a murine model of radiation-induced pneumopathy (Wirsdörfer et al., 2013). Depending on its extracellular concentration, adenosine will act on its receptors (A1, A2A, A2B and/or A3) to reduce the infiltration of inflammatory cells, dampen the release of pro-inflammatory cytokines, and promote tissue repair. Thus, controlled and transient CD73 activity will serve as a critical modulator that helps to mitigate lung damage and inflammation induced by radiotherapy. However, the above-mentioned acute protective effects are distinct from chronically increased CD73/adenosine signaling, which exerts adverse effects by promoting pathologic macrophage polarization, exaggerated matrix deposition chronic pulmonary fibrosis (De Leve et al., 2019b).

It is therefore important to take into account that a combined therapeutic intervention with thoracic irradiation and an anti-CD73 mAB might not only synergize in anti-tumor immunity and limit chronic toxicities (fibrosis), but also synergize in subacute normal tissue toxicities such as radiation pneumonitis to exert stronger adverse effects, if not compensated by other mechanisms (De Leve et al., 2019b). Therapeutic intervention in the CD73/adenosine signaling pathway is therefore a double-edged sword and should be planned with care.

**FIGURE 3:**
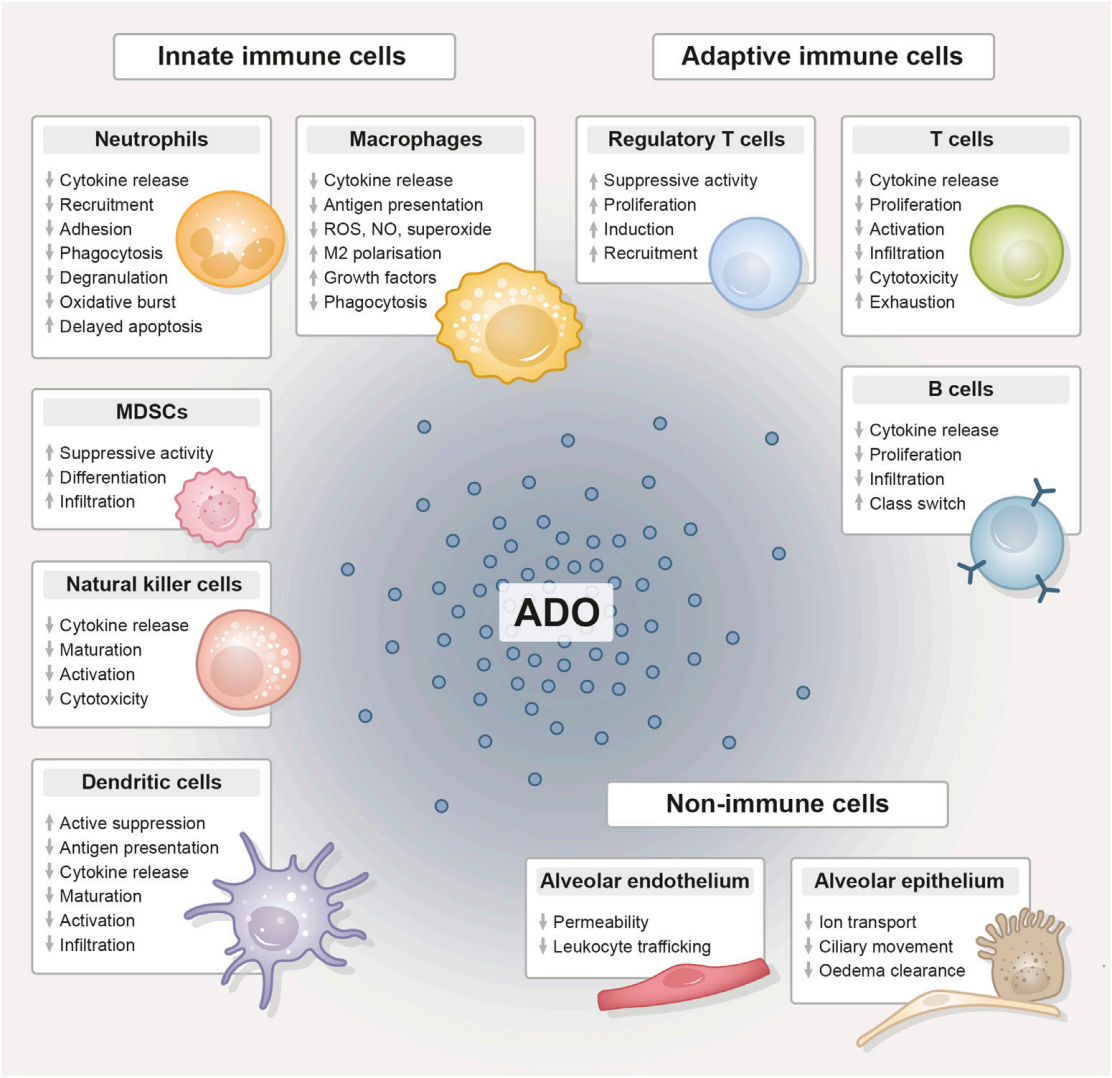
The immunomodulative function of extracellular adenosine. Compilation of the immunomodulative function of extracellular adenosine (ADO), generated from adenosine triphosphate (ATP) by the abundantly expressed CD39/CD73 ectoenzyme machinery, on innate and adaptive immune cell populations and lung-resident non-immune cells. Adenosine unfolds its anti-inflammatory effect primarily by stimulating tolerance-associated cell types, while the functionality and expansion of other immune cells is inhibited. ADO, adenosine; MDSC, myeloid-derived suppressor cell; ROS, reactive oxygen species; NO, nitric oxide.

In the following, we explore overlapping immunomodulatory effects of thoracic radiotherapy and CD73-directed immunotherapy on diverse cell types with relevance to lung injury/pneumonitis. As depicted in Figure 3, CD73/adenosine signaling on immune cells like neutrophils (Cronstein et al., 1985; Cronstein et al., 1990; Richter, 1992; Zalavary et al., 1994; Thiel and Chouker, 1995; Walker et al., 1997; Haskó and Cronstein, 2004; Linden, 2006; McColl et al., 2006; Eltzschig et al., 2008; Säve et al., 2011; Barletta et al., 2012; Petrovic-Djergovic et al., 2012), macrophages (Szabó et al., 1998; Németh et al., 2005; Kreckler et al., 2006; Ramanathan et al., 2007; Csóka et al., 2008; Chen et al., 2009; Buenestado et al., 2010; Ernens et al., 2010; Belikoff et al., 2011), MDSCs (Ryzhov et al., 2011; Sarkar et al., 2023), NK cells (Raskovalova et al., 2006; Young et al., 2018; Chambers and Matosevic, 2019), DCs (Addi et al., 2008; Novitskiy et al., 2008; Wilson et al., 2009; Yang et al., 2010; Silva-Vilches et al., 2018; Wennerberg et al., 2020), T_regs_ (Bopp et al., 2007; Deaglio et al., 2007; Dwyer et al., 2007; Zarek et al., 2008; Nakatsukasa et al., 2011; Ehrentraut et al., 2012; Kinsey et al., 2012; Ohta et al., 2012; Boveda-Ruiz et al., 2013; Schuler et al., 2014), T cells (Erdmann et al., 2005; Lappas et al., 2005; Csóka et al., 2008; Takedachi et al., 2008; Cekic et al., 2013; Leavy, 2013; Cekic and Linden, 2014; Kjaergaard et al., 2018; Leone et al., 2018; Vigano et al., 2019), B cells (Saze et al., 2013; Schena et al., 2013; Przybyla et al., 2018; Jeske et al., 2020) and lung resident non-immune cells like endothelial (Salmi and Jalkanen, 2005; Eckle et al., 2007; Umapathy et al., 2010) and epithelial cells (Lazarowski et al., 1992; Picher et al., 2003; Factor et al., 2007) is related to impaired migration, proliferation, antigen-presentation, pro-inflammatory cytokine and chemokine secretion, as well as impaired cytotoxicity and other anti-tumor responses and would therefore counteract radiation-induced pneumonitis. The therapeutic intervention with an anti-CD73 therapy has therefore the potential to reduce adenosine production and signaling and as a consequence limit the mentioned changes in the depicted cells. In the irradiated (damaged) normal tissue, pro-inflammatory responses are consequentially not dampened to a similar extent and, dependent on the severity of damage or immune response, can result in exaggerated inflammation and stronger radiation pneumonitis. Unfortunately, data on therapeutic targeting of CD73 in the context of acute and subacute radiation-induced lung toxicities is very limited. The available studies that investigate the role of CD73/adenosine targeting mostly show the therapeutic effects on tumor responses, thus we summarize here the major immunomodulatory actions of CD73/adenosine and its intervention and discuss a potential impact on toxic side effects in the lungs in the following paragraph.

For antigen-presenting cells like DCs, it was shown in a murine breast cancer model that an anti-CD73 therapy combined with radiotherapy restored conventional DC (cDC) activity and their infiltration into the tumor, enhanced the CD8^+^/T_reg_ ratio and improved tumor control (Wennerberg et al., 2020). However, the positive reactivation or cancellation of suppression could have negative consequences in the irradiated normal tissue. The irradiated and inflamed lung microenvironment also influences the phenotype of lung DCs. Dependent on the micromilieu, DCs can be differentiated into TNF-DCs, IFN-DCs, (thymic stromal lymphopoietin) TSLP-DCs, IL-15-DCs, chemokine C-C-motif ligand 19 (CCL19)-DCs, or chemokine CXC-motif ligand 4 (CXCL4)-DCs by TNF-α, IFNγ, IL-15, IL-10, CCL19, and CXCL4, respectively (Liu et al., 2023). For instance, TSLP-DCs secrete high levels of type 2 cytokines and TNFα (Tormo and Gauchat, 2013). IFNγ-DCs increase the release of IL-12, which facilitates an efficient T-cell response (Gagliostro et al., 2016). CXCL4-DCs promote the generation of IFNγ and IL-4 as well as the growth of autologous CD4^+^ and CD8^+^ T cells (Silva-Cardoso et al., 2020; Gowhari Shabgah et al., 2021). Moreover, monocyte-derived inflammatory DCs (inf-DC, also called mo-DC) will also be present in the irradiated lung tissue, which derive from circulating Ly6C^high^ monocytes that are considered to be the direct precursors of inf-DCs (Merad et al., 2013). These inf-DCs produce high amounts of TNFα and NO, thus also called “**T**NFα and **i**NOS **p**roducing” Tip-DC (Serbina et al., 2003; Cook and MacDonald, 2016). Thus, we speculate that, dependent on the individual lung milieu, anti-CD73 therapy may impact specific DC subsets and thereby inducing stronger pro-inflammatory cytokine and ROS production and enhancing the risk for radiation pneumonitis.

The same holds true for other innate immune cells like neutrophils and macrophages. A modulation of CD73 activity can alter macrophage functions by switching M1 and M2 phenotypes and also downregulate neutrophil activity (Haskó and Cronstein, 2004; Eltzschig et al., 2008; Yegutkin et al., 2011; Zanin et al., 2012; Ponce et al., 2016). As a consequence of targeted anti-CD73 therapy, both cell types could show a more pronounced pro-inflammatory phenotype and a series of cytokines, such as TNFα, IL-1β, IL-6, and TGFβ, could be released and enhance pneumonitis (Yarosz and Chang, 2018).

NK cells, important mediators of tumor-killing, have low levels of CD73 expression. Nevertheless, this expression is significant in tissues that have been invaded by tumors, indicating that NK cells may be able to inhibit the immune system by producing adenosine, if certain environmental conditions are met (Chatterjee et al., 2014). Adenosine generated by CD73 mainly inhibits NK cell activities via A2A (Raskovalova et al., 2006; Wang and Matosevic, 2018), thus leading to impaired maturation, activation, and cytotoxic potential of NK cells (Raskovalova et al., 2006; Beavis et al., 2013; Hatfield et al., 2015; Young et al., 2016; Chambers et al., 2018); in addition, NK cells in the tumor microenvironment undergo transcriptional reprogramming and upregulate IL-10 production (Neo et al., 2020). A study from Wang et al. showed in a murine model of radiation pneumonitis that radiation pneumonitis was accompanied by an accumulation of NK cells and a decline in their IFNγ and granzyme B production. Although an involvement of the purinergic pathway was not analyzed, the authors showed in a pathway enrichment analysis using the KEGG database differentially expressed genes for “nucleotide binding” and “ATP-binding” (Wang et al., 2023). Thus, we speculate that NK cells indeed can be negatively modulated by CD73/adenosine in the normal tissue and also that a therapeutic CD73 targeting could boost their activation and cytotoxic potential.

Besides a modulation of innate immune responses, also adaptive immune responses would be affected by an anti-CD73 therapy. We and others already highlighted that lymphocytes are increased in lung cancer patients and experimental mice and that recruited T lymphocytes release pro-inflammatory TNFα, IFNγ, IL-2, and lymphotactin during the early pneumonitic phase, which lasts from week 3–12 post radiation in a murine model (Schaue and McBride, 2012; Schaue et al., 2012; Wirsdörfer and Jendrossek, 2016; Lierova et al., 2018; Zhou P. et al., 2020). Following thoracic irradiation in mice, in addition to a typical T_h_1 response, a pro-inflammatory T_h_17-dominant response was observed. Three weeks after radiation, there was an increase in the cytokine levels of IL-16, IL-17, IL-23, and IL-27, which might contribute to tissue damage and chronic inflammation (Cappuccini et al., 2011). Since CD73/adenosine signaling dampens these adaptive responses, an anti-CD73 therapy would also in this immune compartment reduce counteracting anti-inflammatory signaling, promote T_h_1 and T_h_17 responses and thus have the potential to foster pneumonitis development.

In the immune cell compartment, also immunosuppressive cell types like MDSCs or T_regs_ would be directly altered by an anti-CD73 therapy. The blockage of purinergic signaling and reduction in adenosine would lead to reduced recruitment and accumulation of these cells (Zarek et al., 2008; Mandapathil et al., 2010; Ryzhov et al., 2011; Iannone et al., 2013), thus unbalancing negatively regulated immune responses towards enhanced inflammation.

Among the resident lung cells, the alveolar epithelium plays a major role in modulating and inducing immune responses and maintaining homeostasis (Whitsett and Alenghat, 2014). In AEC cells, a PRR response to viral/bacterial components and allergens induces the induction of type I and type III interferons (Wang et al., 2009; Slater et al., 2010), as well as other cytokines (IL-1α, IL-1β, IL-6, IFNγ, TNFα, IL-33, IL-25) and chemokines (CXCL8, CCL2, CCL3, CC4, and CCL5) to further promote immune responses (Clemans et al., 2000; Glaser et al., 2019; Blanco-Melo et al., 2020; Vanderheiden et al., 2020). Thus, we speculate that also DAMPs released after radiation damage can, via the same PRRs, induce pro-inflammatory signaling. In addition, CD73 helps epithelial cells to maintain a state of homeostasis unique to the lung tissue to regulate respiratory processes. On the surface of the respiratory system’s airway epithelial cells, CD73 is the main source of extracellular adenosine. Extracellular adenosine regulates mucociliary clearance and ion exchange, including chloride, and also reduces endothelial permeability, preserving the integrity of the tissue barrier (Lazarowski et al., 1992; Picher et al., 2003; Colgan et al., 2006; Eckle et al., 2007). Moreover, Factor et al. (2007) demonstrated that activation of A2A can promote alveolar epithelial sodium transport, which is beneficial to pulmonary oedema clearance. Thus, an anti-CD73 therapy could promote epithelial-induced pro-inflammatory signaling, enhanced vascular leakage and reduced pulmonary oedema clearance with a potential contribution to enhanced normal tissue damage and pneumonitis.

Similar observations have been made in lung endothelial cells: CD73 is involved in orchestrating leukocyte trafficking in response to chemotactic stimuli (Eltzschig et al., 2004; Salmi and Jalkanen, 2005; Linden, 2006) and mice lacking CD73 display an increased adhesion of leukocytes to the vascular endothelium (Eltzschig et al., 2004; Dwyer et al., 2007; Takedachi et al., 2008; Petrovic-Djergovic et al., 2012). Specifically, the reduced synthesis of adenosine in these animals was linked to elevated endothelial activation, recruitment of monocytes, and aggregation of platelets, indicating a crucial function for these enzymes in the pathophysiology of vascular inflammation (Koszalka et al., 2004; Zernecke et al., 2006). We therefore speculate that also in endothelial cells a therapeutic intervention in CD73/adenosine dynamics could favor the development of pneumonitis.

Besides the aforementioned cell types, MSCs in both the tumor and normal lung tissue, as well as MSC-derived EVs, express CD73 and could thus be modulated by anti-CD73 therapy. At present, the function of MSC-derived CD73 in radiation pneumonitis specifically is unknown and warrants further investigation. However, tumor-resident MSCs mediate immunosuppression via the CD73/adenosine axis, leading to a reduced activity of cytotoxic T cells and NK cells. In radiation-pneumonitis similar regulatory mechanisms could apply to counteract lung inflammation, which would be disabled by early targeting of CD73 (Gottschling et al., 2013; Ramos et al., 2016; Tan et al., 2019; Kou et al., 2022).

Finally, we emphasize again, that an anti-CD73 therapy could clearly inhibit the immunosuppressive and pro-fibrotic effects mediated by CD73 in radiation-induced lung fibrosis. As mentioned, CD73 expression and activity typically increases in response to radiation, and own work revealed that CD73-dependent chronic accumulation of adenosine also contributes to pathologic macrophage M2 polarization and progression towards lung fibrosis (Wirsdörfer et al., 2013; De Leve et al., 2017). Targeting of CD73 reduced adenosine production, thereby enhancing the immune system’s ability to clear damaged cells and decrease fibrotic processes, whilst also promoting immune-mediated anti-tumor responses (De Leve et al., 2019a). The enhanced clearance of damaged cells results in decreased inflammation and potentially mitigates the progression to pulmonary fibrosis. Preclinical studies suggest that an anti-CD73 therapy can alleviate symptoms and improve lung function in radiation-induced fibrosis, highlighting its potential as a therapeutic approach to enhance patient outcomes following thoracic radiation therapy (De Leve et al., 2019a).

To summarize, a CD73-directed immunotherapy could drive a palette of resident non-immune cells as well as immune cells in the irradiated lung towards an inflammatory phenotype or signaling that might contribute to the development of radiation-pneumonitis, whilst attenuating chronic effects that promote progression towards radiation-induced lung fibrosis.

For the treatment of stage III unresectable NSCLC, however, a multi-combinatorial approach with additive targeting of CD73 to the established *PACIFIC* regimen (concurrent chemo-radiotherapy with anti-PD-L1 consolidation) is evaluated in clinical trials (*COAST* and *PACIFIC-9*). Since each treatment in itself harbors the risk of triggering irAEs, it is assumed that combinatorial approaches have overlapping or even additive effects and may therefore increase the risk for both, the occurrence and severity of normal tissue toxicities. In the following, we compare incidence rates of (radiation) pneumonitis from the respective clinical studies to evaluate the risk for enhanced irAEs in the clinical setting.

## 4 Clinical evidence for ICI-augmented radiation pneumonitis in the treatment of advanced NSCLC

About one-third of all NSCLC diagnosed patients are in stage III and the majority of these cases are unresectable. Based on the findings of the phase III *PACIFIC* trial (NCT02125461), platinum-based cCRT with the anti-PD-L1 mAB Durvalumab as consolidation therapy for 1 year is the current standard of care for patients with unresectable stage III NSCLC (Antonia et al., 2017; 2018; Cortiula et al., 2022).

Although the addition of adjuvant Durvalumab immunotherapy has durable benefits for overall (OS) and progression-free survival (PFS), the 5-year OS and PFS rates of the *PACIFIC* trial remain low at 42.9% and 33.1%, respectively (33.4% OS and 19.0% PFS in the placebo arm) (Faivre-Finn et al., 2021; Spigel et al., 2022). Subsequent real-world studies imply that the actual survival rates outside of the idealized trial setting are even lower in certain cohorts (Sankar et al., 2022). The significant proportion of non-responding and relapsing patients stresses the demand for novel treatment concepts. Consequentially, various clinical trials followed *PACIFIC* to explore adapted treatment concepts, such as the use of alternative PD-L1-targeting antibodies (e.g., Atezolizumab, Nivolumab, Ipilimumab and Pembrolizumab), sequential chemo-radiotherapy (sCRT) or induction and concurrent ICI treatment (Cortiula et al., 2022).

Recent innovative treatment strategies include targeting of the purinergic signaling system with the anti-CD73 mAB Oleclumab as an additive to Durvalumab consolidation treatment (Herbst et al., 2022). In the following, we review the incidence rates of (radiation) pneumonitis with the *PACIFIC* regimen and novel treatment concepts involving CD73-targeted immunotherapy (Table 1) (Taugner et al., 2021; Antonia et al., 2017; Faehling et al., 2020; Jung et al., 2020; Herbst et al., 2022; Sugimoto et al., 2022; Akkad et al., 2023; Girard et al., 2023; Barlesi et al., 2024; Sato et al., 2024). However, it should be noted that not all studies mentioned here differentiate between pneumonitis and radiation pneumonitis and moreover pneumonitis is occasionally used as a collective term that also includes interstitial lung disease (ILD) or pulmonary fibrosis (Girard et al., 2023). Thus, based on these variable criteria, a direct comparison of the pneumonitis incidence between patient cohorts is challenging.

**TABLE 1:** Pneumonitis rates of combined chemo-radio-immunotherapies in stage III NSCLC.

Clinical trial (ID)	Duration	Enrollment	Study design	Treatment sequence	Treatment arms	P/RP^a^ any grade	P/RP^a^ grade 3 or higher	Drug discontinuation due to P or RP	Reference
*PACIFIC* regimen									
PACIFIC NCT02125461	2014 -2023 completed	713	Randomized phase III	cCRT with adjuvant ICI	cCRT + Placebo	**24.8%**	**2.6%**	^b^ **4.3%**	Antonia et al. (2017)
					cCRT + Durv	**33.9%**	**3.4%**	^b^ **6.3%**	
*PACIFIC* regimen: selected real-world cohorts									
PACIFIC-R NCT03798535	2018–2024 active, not recruiting	1,399	Retrospective	cCRT or sCRT with adjuvant ICI	cCRT or sCRT + Durv	**18.3%** ^c^	**3.3%** ^c^	^b^ **14.7%** 5.2% temp. 9.5% perm.	Girard et al. (2023)
Patient cohort Samsung Medical Center, Korea	2019–2019	61	Retrospective	cCRT with adjuvant ICI	cCRT	**RP 37.5%**	**RP 2.5%**	NA	Jung et al. (2020)
					cCRT + Durv	**RP 81%**	**RP 14.3%**	**42.9%** 23.8% temp. 19% perm.	
Patient cohort Yokohama Municipal Citizen’s Hospital, Japan	2013–2022	150	Retrospective	cCRT with adjuvant ICI	cCRT	**26.3%** out-of-filed P 4.9% in-filed P 20.9%	**12.6%**	NA	Sato et al. (2024)
					cCRT + Durv	**41.9%** out-of-field P 20.2% in-field P 20.2%	**10.5%**	^b^ **50.7%** 30.1% temp. 20.6% perm.	
Patient cohort 56 centers, German EAP	2017–2018	126 (incl. 7 stage IV patients)	Retrospective	Mostly sCRT with adjuvant ICI	cCRT + Durv	**15%**	**8.7%**	NA	Faehling et al. (2020)
Patient cohort single center, Germany	2018–2020	26	Prospective	cCRT or sCRT with adjuvant ICI	cCRT or sCRT + Durv	**15.3%**	**15.3%**	^b^ **15.3%**	Taugner et al. (2021)
Patient cohort multi center, veterans	2017–2020	284 (incl. 1 stage I, 21 stage II and 35 unknown stage patients)	Retrospective	cCRT with adjuvant ICI	cCRT + Durv	**21.4%**	**12.7%**	^b^ **21.4%** 5.2% temp. 16.2 perm.	Akkad et al. (2023)
Patient cohort multi center, baseline G1 RP	2019	35	Prospective	cCRT with adjuvant ICI	cCRT + Durv	**31%**	**3%**	NA	Sugimoto et al. (2022)
adapted *PACIFIC* regimen: dual ICI consolidation									
COAST NCT03822351	2018–2023 completed	189	Randomized Phase II	cCRT with adjuvant ICI	cCRT + Durv	**P 16.7%**/**RP 4.5%**	**P 0%**/**RP 1.5%**	^b^ **6.1%**	Herbst et al. (2022)
					cCRT + Durv + Olec	**P 18.6%**/**RP 11.9%**	**P 0%**/**RP 0%**	^b^ **5.1%**	
					cCRT + Durv + Mona	**P 16.4%**/**RP 4.9%**	**P 1.6%**/**RP 0%**	^b^ **4.9%**	
PACIFIC-9 NCT05221840	2022–2030 recruiting	(999)	Randomized Phase III	cCRT with adjuvant ICI	cCRT + Durv + Placebo	NA	NA	NA	Barlesi et al. (2024)
					cCRT + Durv + Olec	NA	NA	NA	
					cCRT + Durv + Mona	NA	NA	NA	

^a^ Pneumonitis (P)/Radiation pneumonitis (RP) definition varies between cohorts.

^b^ P/RP was the most frequent adverse event-related cause for discontinuation.

^c^ A single patient could present with multiple events of different severity.

Bold values indicate overall incidences.

### 4.1 Pneumonitis incidence with the *PACIFIC* regimen

The *PACIFIC* trial (NCT02125461) is an international, randomized, phase III clinical trial, which enrolled 713 patients with stage III, locally advanced, unresectable NSCLC from 2014–2023 to compare Durvalumab consolidation to placebo after platinum-based cCRT (Antonia et al., 2017; 2018). The combined incidence rate of any-grade pneumonitis and radiation pneumonitis reported for the *PACIFIC* trial was 24.8% (2.6% for grade 3–4) in the placebo arm compared to 33.9% (3.4% for grade 3–4) in the Durvalumab arm. Overall, treatment-related pneumonitis and radiation pneumonitis were considered to be within reasonable limits and manageable, but nonetheless constituted the most frequent adverse event requiring treatment discontinuation (4.3% in placebo arm and 6.3% in Durvalumab arm) (Antonia et al., 2017).

The *PACIFIC* trial demonstrated a sustained benefit of Durvalumab consolidation therapy on OS and PSF. Yet, the majority of NSCLC patients in clinical practice does not meet the idealized eligibility criteria of the *PACIFIC* trial, but might nonetheless benefit from adjuvant immunotherapy. For instance, patients with low performance status, autoimmune disease or ≥ Grade 2 pneumonitis from prior chemo-radiotherapy, as well as patients receiving radiotherapy with a total radiation dose above 66 Gy, a mean dose to the lung (MLD) of 20 Gy or more or a V20 (lung parenchyma volume receiving ≥ 20 Gy) of 35% or more were excluded from the study, implying that the incidence of pneumonitis and radiation pneumonitis might be underestimated in this setting (Antonia et al., 2017; Faehling et al., 2020; Jung et al., 2020). Therefore, safety assessment of Durvalumab consolidation therapy in real-world patient cohorts is more decisive. On the basis of the *PACIFIC* trial, the retrospective *PACIFIC-R* trial (NCT03798535) evaluated the efficacy and safety of the *PACIFIC* regimen in 1,399 patients with unrespectable, stage III NSCLC that received Durvalumab after cCRT or sCRT through an early access program (EAP). In this real-world data set, any-grade pneumonitis occurred in 18.3% of the patients (3.3% grade 3 or higher), while the percentage of patients that had to temporarily or permanently discontinue Durvalumab treatment due to pneumonitis was higher (14.7%) than reported for *PACIFIC* (Girard et al., 2023). Although the eligibility criteria for the *PACIFIC-R* trial were less restrictive, the incidence and severity of pneumonitis in this real-world patient cohort were thus still comparable to the original *PACIFIC* trial. Yet, other exemplary retrospective studies on real-world stage III NSCLC patient cohorts treated with the *PACIFIC* regimen have reported much higher pneumonitis rates. For instance, Jung et al. specifically investigated the occurrence of radiation pneumonitis in a Korean patient cohort and reported that 81.0% of patients treated with the *PACIFIC* protocol developed any-grade radiation pneumonitis, compared to 37.5% of patients receiving cCRT without Durvalumab consolidation therapy. For grade 3 or higher radiation pneumonitis, the incidence rates were 14.3% and 2.5% in the respective groups. Among high MLD ≥ 20 Gy and a V20 of 35% and above, Durvalumab was identified as the most relevant risk factor for radiation pneumonitis and significantly associated with reduced radiation pneumonitis-free survival (Jung et al., 2020). In a Japanese patient cohort, Durvalumab consolidation therapy after cCRT was reported to increase the occurrence of any-grade pneumonitis outside of the radiation field (4.9% in control arm and 20.2% in Durvalumab arm), while the incidence rate of in-field-pneumonitis was similar (20.9% in control arm and 20.2% in Durvalumab arm). However, the occurrence of higher-grade pneumonitis was comparable between both arms. Further exemplary patient cohorts are listed in Table 1.

### 4.2 Pneumonitis incidence with the *PACIFIC* regimen and additive targeting of CD73

Although pneumonitis rates reported for the *PACIFIC* regimen are variable, clinical trial and real-world studies demonstrate an increased risk for pneumonitis and radiation pneumonitis for chemo-radiotherapies when combined with adjuvant Durvalumab monotherapy (Table 1). However, since it is apparent across studies that primarily the risk of low-grade (radiation) pneumonitis, but not the incidence of severe cases is increased, irAEs associated with Durvalumab are mostly considered tolerable, given the improved chances of survival (Antonia et al., 2017; Jung et al., 2020; Chen et al., 2023; Girard et al., 2023; Sato et al., 2024). In order to further improve the efficiency of the *PACIFIC* regimen, additive targeting of CD73 in the Durvalumab consolidation phase is being considered and already evaluated in clinical trials. This raises the question whether further dysregulation of the immune system by addition of a second ICI could significantly increase the risk of irAEs like pneumonitis or radiation-pneumonitis.

Chemo-radiotherapy with dual ICI therapy was assessed in the *COAST* study (NCT03822351), an international, randomized, phase II clinical trial that enrolled 189 patients with unresectable stage III NSCLC and was completed in 2023. Apart from the anti-CD73 mAB Oleclumab, Durvalumab was alternatively also combined with the mAB Monalizumab that targets natural killer group protein 2A (NKG2A), a human leukocyte antigen (HLA)-E-binding inhibitory receptor expressed on the surface of NK and T cells. Both treatment arms with dual ICI consolidation therapy achieved superior response and survival rates, compared to consolidation with Durvalumab monotherapy (Herbst et al., 2022). Overall, safety profiles were reported to be similar between mono and dual ICI treatments. However, the any-grade pneumonitis incidence was 16.4% in the Durvalumab arm, compared to 18.6% in the Durvalumab plus Oleclumab arm. The incidence of any-grade radiation pneumonitis in particular diverged even more between these arms with 4.5% the Durvalumab and 11.9% in the Durvalumab plus Oleclumab cohort. In contrast, the occurrence of pneumonitis and radiation pneumonitis upon dual ICI consolidation therapy with Durvalumab plus Monalizumab was similar as with Durvalumab monotherapy, potentially because the immunomodulative range of CD73 is higher than that of NKG2A. Yet, in line with the original *PACIFIC* regimen, the increased incidence of (radiation) pneumonitis in the Durvalumab plus Oleclumab arm is only apparent for low-grade (radiation) pneumonitis, but not for grade 3-4 (radiation) pneumonitis. As reported for the *PACIFIC* regimen, pneumonitis was likewise the most common adverse event causing treatment discontinuation in the *COAST* trial, but with no difference between the treatment arms (6.1% in the Durvalumab arm and 5.1% in the Durvalumab plus Oleclumab arm). Among the four drug-related deaths reported in this trial, pneumonitis was also the most common cause, with two patients in the Durvalumab arm dying from pneumonitis and radiation pneumonitis, respectively, and one patient in the Durvalumab plus Oleclumab arm dying from pneumonitis (Herbst et al., 2022).

At present, cCRT followed by dual ICI consolidation therapy (Durvalumab plus Oleclumab and Durvalumab plus Monalizumab) is further evaluated in the ongoing *PACIFIC-9* study (NCT05221840), an international, randomized, placebo-controlled, phase III clinical trial with an anticipated enrolment of 999 subjects (Remon et al., 2022; Barlesi et al., 2024). The upcoming results of the *PACIFIC-9* trial, as well as future real-world data will show whether *COAST* safety profiles of chemo-radiotherapy with combined PD-L1- and CD73-targeted immunotherapy can be confirmed in a larger cohort.

## 5 Conclusion

Therapeutic targeting of immune checkpoint inhibitors has demonstrated great benefit for treatment outcome in NSCLC and other solid tumor entities and treatment efficacy can be further augmented when ICIs are combined with conventional immune-activating interventions like radiotherapy or definitive chemo-radiotherapy. For stage III unresectable NSCLC, consolidation immunotherapy targeting PD-L1 has become a new standard of care in patients without disease progression after concurrent platinum-based chemo-radiotherapy (*PACIFIC* regimen). As novel therapeutics targeting pivotal immunosuppressive immune checkpoints in the tumor microenvironment are rapidly being developed, navigating the risks and rewards of these increasingly complex multi-combinatorial treatment strategies is exceptionally challenging.

In this review, we explored how the therapeutic inhibition of the immunosuppressive CD73/adenosine pathway and the resulting altered ATP/adenosine dynamics in the lung tissue could exacerbate the development of radiation pneumonitis in patients receiving thoracic chemo-radiotherapy combined with ICI-based immunotherapy. Though interactions between ICI and chemo-radiotherapy have so far primarily been investigated in the context of treatment efficacy, we extrapolated the results on therapy-induced changes in the immune repertoire to normal tissue effects and the development of (radiation) pneumonitis. We assume that analogue immune mechanisms may apply in the development of radiation pneumonitis and thus conclude that inhibition of CD73 and the consequentially diminished conversion of extracellular adenine-nucleotides into adenosine disrupts a pivotal immunosuppressive mechanism in both, the tumor environment and the co-irradiated normal lung tissue. In the irradiated normal tissue, inhibition of immunosuppressive CD73/adenosine signaling might at least partially impair inflammatory resolution and thereby potentially escalate pro-inflammatory responses of radiotherapy. These effects may ultimately increase the risk for subacute radiation pneumonitis, particularly when tissue tolerance to the inflammatory insult from chemo-radiotherapy is additionally challenged by inhibition of PD-1/PD-L1 signaling.

The benefits of such multi-combinatorial treatment regimens are expected to outweigh the potential risks for most treatment-responsive patients, although the consequences of irAEs can be fatal for the individual. In the recently completed *COAST* trial, additive targeting of CD73 increased the risk for any grade radiation pneumonitis from 4.5% with anti-PD-L1 alone, to 11.9% with the co-treatment. Yet, as in other radio-immunotherapy trials, no such increase was observed for higher grade radiation pneumonitis; therefore, the increased risk of combined toxicity is considered to be manageable. Nevertheless, it has to be taken into account that (radiation) pneumonitis is so far the most frequent cause for treatment discontinuation, whereas treatment continuation can cause pneumonitis recurrence in sensitive patients. Regarding the overall consequences of treatment discontinuation due to pulmonary toxicities, some studies report negative implications on survival and metastasis, while others did not identify such a link with worse outcomes (Xu et al., 2022; Akkad et al., 2023). As data on the *PACIFIC* regime with additive CD73 targeting from the ongoing *PACIFIC-9* trial is still pending, it remains to be ascertained if the incidence rates of radiation pneumonitis reported to date can be confirmed in a larger cohort. Furthermore, the impact of multitargeting therapies on long-term benefit and quality of life, e.g., with regard to incidence of recall phenomena as well as incidence and severity of pulmonary fibrosis, should also be evaluated.

## 6 Future challenges

At present, investigations on the effects of combined radio-immunotherapies primarily focus on treatment efficacy. In view of the increasing clinical use of combined treatments and the rapid clinical progress of dual or multi-targeting radio-immunotherapy, broad pre-clinical, co-clinical and clinical investigations assessing heterogeneity in dynamic local and systemic normal tissue effects are urgently needed in order to define potential new or increased pulmonary toxicities, underlying mechanisms and associated risk factors. The findings from these studies are fundamental to implement novel ICIs (e.g., anti-CD73) into established regimens and determine individual predictors for susceptibility to irAEs. Ultimately, the biggest future challenge for optimized radio-immunotherapies will be to align the treatment design (6.1) with individual patient factors (6.2), hence paving the way for individualized treatment concepts (personalized medicine).

### 6.1 Considerations for treatment design

#### 6.1.1 General treatment design

Multi-combinatorial radio-immunotherapy concepts require careful fine-tuning of each individual treatment parameter to account for potentially overlapping or synergistic immunomodulatory effects, including physical (radiotherapy dose and volume, fractionation, radiation quality), biological/mechanistic (choice of ICI, ICI interactions, treatment sequence and timing) and clinical (patient-related factors) aspects. For example, despite broad knowledge on dose-volume constraints, suitable fractionation schemes for radiotherapy and appropriate schedules for combinations with conventional chemotherapy, these parameters need to be redefined for each novel combinatorial approach. Furthermore, it has been acknowledged that the timing and sequence of combined radio-immunotherapy, are critical determinants of treatment efficacy and presumably also for the development of toxicities (Aliru et al., 2018; Anscher et al., 2022). The implementation of a novel ICI (e.g., anti-CD73) into established radio-immunotherapy protocols (e.g., *PACIFIC* regimen) thus needs to be coordinated with the radiation plan, the administration of chemotherapeutics and, in the case of dual-ICI strategies, the co-targeting of another immune checkpoint (e.g., PD-L1). Here, the double-edged effect of a maximized immune activation, which is sought for an optimal treatment response, but at the same time poses the highest risk for the development of irAEs, is a particular challenge.

#### 6.1.2 ICI choice

Regarding ICI-based immunotherapies, it needs to be considered that not only the choice of the targeted pathway, but also the choice of the distinct component of the respective pathway (e.g., ligand versus receptor targeting) will determine the immune response. In this regard, it has already been shown that therapeutic targeting of the PD-1 receptor is associated with a higher incidence of irAEs than targeting of its ligand PD-L1, which is potentially based on the fact that targeting of PD-L1 spares PD-1/PD-L2 interactions (Pillai et al., 2018; Shankar and Naidoo, 2018). Likewise, it can be expected that targeting of CD73 exerts different effects than inhibition of other components of the purinergic system (e.g., drug-targeting ectonucleotidases involved in ATP conversion to adenosine versus adenosine receptors).

#### 6.1.3 ICI interactions

Radio-immunotherapies with dual-ICI targeting, as currently evaluated for PD-L1 and CD73/NKG2A in the *COAST* and *PACIFIC-9* trials, will also increase the complexity of the resulting immune response in the co-irradiated normal tissue and thus further complicate the risk prediction and management of potential new or increased irAEs. Herein, not only potential synergistic effects and overlapping pathways between the radio- and ICI-therapy, but also interactions between two distinct immune checkpoints or alternative compensatory mechanisms need to be considered. For the CD73/adenosine system and the PD-1/PD-L1 pathway, a direct interaction has mainly been described for T cells. For instance, adenosine-mediated A2A signaling was shown to augment the expression of PD-1, but not CTLA-4, on CD8^+^ T cells and also on T_regs_ (Allard et al., 2013). Complementary, blockade of PD-1 on tumor-infiltrating CD8^+^ T cells resulted in an increased A2A expression on these cells (Beavis et al., 2015). However, since components of PD-1/PD-L1 and CD73/adenosine signaling pathways are also expressed on various other immune cells as well as non-immune cells in the lung, more complex interactions can be expected from dual-ICI treatment.

### 6.2 Considerations for patient stratification

#### 6.2.1 Individual risk factors

As the complexity of multi-combinatorial treatment plans increases, the preceding risk assessment consequentially should reflect an equal degree of complexity to optimize patient stratification and ensure treatment safety. The acknowledgement of individual risk factors, including age, sex, general health status, pre-existing illnesses, pulmonary and cardiac constitution, genetic susceptibility factors, treatment tolerance, the immune status and the microbiome, as well as the presence of autoimmune diseases (Chen et al., 2021; Yamaguchi et al., 2022; Moratiel-Pellitero et al., 2024), that could predispose sensitive patients to irAEs, will inevitably become increasingly important, irrespective of the immune checkpoints targeted. The role of baseline expression of targeted immune checkpoints as a patient-specific risk factor for the occurrence or severity of irAEs in combined radio-immunotherapies remains unclear. Tumor-specific expression of CD73 has been implicated in cancer progression and therapeutic outcomes, with expression levels influenced by both genetic and environmental factors. In this context, higher CD73 expression has been associated with factors such as female gender, non-smoking status, hypoxia, and specific oncogenes (e.g., KRAS and EGFR) (Inoue et al., 2017; Cao et al., 2021; Kowash and Akbay, 2023). Dynamic CD73 alterations have also been observed in other pathophysiological conditions (e.g., autoimmunity, infections, cardiovascular and atherosclerotic diseases), as well as in response to hormonal changes, with unknown consequences for occurrence of irAE in combined radio-immunotherapies (Deaglio and Robson, 2011; Nikolova et al., 2011; Bönner et al., 2012; Antonioli et al., 2013; Lee et al., 2021; Ndzie Noah et al., 2023). Whether CD73 expression in normal tissues is affected by similar factors and whether it constitutes a patient-specific risk factor for irAEs remains an area for further investigation.

#### 6.2.2 Risk prediction technology and biomarker

Novel risk prediction technologies will be essential to meet those more demanding requirements. In this context, promising results to predict the occurrence of radiation pneumonitis have, for instance, been achieved in the field of CT-based radiomics, which can certainly be further refined in the AI era (Cunliffe et al., 2015; Krafft et al., 2018; Hope et al., 2021). In addition to such imaging-derived biomarkers, there is likewise an unmet need for sound molecular biomarkers predicting the individual risk of developing radiation pneumonitis. For example, significant decreases in absolute lymphocyte counts, increases in the neutrophil to lymphocyte ratio (NLR) and early variations in T cell subsets in the peripheral blood have been associated with both, ICI pneumonitis and radiation pneumonitis (Lee et al., 2018; Zhou P. et al., 2020; Lin et al., 2021). First clinical observations in peripheral blood of irradiated esophageal cancer patients support a potential predictive value of a radiotherapy-induced increased T_h_17/T_reg_ ratio at 5 weeks after the onset of radiotherapy for occurrence of radiation pneumonitis (Wang et al., 2017). Nevertheless, there is still a gap of knowledge how the dynamic interplay between T_h_17 cells, T_regs_ and other T cell subsets, innate immune cells, and parenchymal cells in the irradiated lung environment promotes progression to radiation pneumonitis and potentially pulmonary fibrosis. As current pre-clinical studies primarily investigate the effects of combined radio-immunotherapy with regard to treatment efficacy, mechanistic investigations with a focus on new or increased immune-related adverse effects or other toxicities are rare. So far, only single studies analyzed increased early pulmonary toxicity of thoracic irradiation with concurrent administration of PD-1 targeted ICI therapy. In these studies, acute pulmonary toxicity was associated with a disturbance of the T cell compartment and increased levels of IL-17A or IL-6 and TGFβ (Sheng et al., 2021; Geng et al., 2022). Since radiotherapy- and combinatorial radio-immunotherapy-induced immune changes are dynamic, it should be explored if basal differences or pathology-associated dynamic changes of specific innate immune cells or lymphocyte subsets, associated cytokines/chemokines, soluble forms of immune checkpoints (e.g., soluble CD73 or PD-1) (Yegutkin, 2008; Antonioli et al., 2013) or general inflammation markers are suited as prognostic or diagnostic biomarker. Because of the assumed multifactorial disease pathogenesis, it is highly likely that not a single factor may be suited to predict the individual risk for toxicity, but rather a combination of markers.

#### 6.2.3 Monitoring and guidelines

In addition to a thorough risk profile assessment before treatment, a regular and frequent monitoring of the patient during and after treatment is likewise advisable, particularly as radiation pneumonitis is a late-occurring complication, challenging to discriminate from ICI- or infection-induced pneumonitis or other similarly presenting conditions, and can eventually progress to fatal pulmonary fibrosis (Jung et al., 2020; Guberina et al., 2023). In this context, attention should be paid on including samples that are accessible from patients under therapy (e.g., peripheral blood, bronchioalveolar lavage).

The definition of relevant diagnostic and prognostic biomarkers indicating an increased individual risk for overlapping toxicities and their translation to clinical application requires broad efforts of multiple research groups. However, on the long term, such an effort may allow for an individualized biomarker-driven treatment planning in the future (Boerma et al., 2022). Considering the complexity of such efforts, future treatment decision-making and monitoring would immensely benefit from the establishment of defined risk assessment guidelines.

## References

[B1] Addi A. B. Lefort A. Hua X. Libert F. Communi D. Ledent C. (2008). Modulation of murine dendritic cell function by adenine nucleotides and adenosine: involvement of the A2B receptor. Eur. J. Immunol. 38, 1610–1620. 10.1002/eji.200737781 18465770

[B2] Akkad N. Thomas T. S. Luo S. Knoche E. Sanfilippo K. M. Keller J. W. (2023). A real-world study of pneumonitis in non-small cell lung cancer patients receiving durvalumab following concurrent chemoradiation. J. Thorac. Dis. 15, 6427–6435. 10.21037/jtd-22-1604 38249904 PMC10797388

[B3] Ålgars A. Karikoski M. Yegutkin G. G. Stoitzner P. Niemelä J. Salmi M. (2011). Different role of CD73 in leukocyte trafficking via blood and lymph vessels. Blood 117, 4387–4393. 10.1182/BLOOD-2010-11-321646 21346249

[B4] Aliru M. L. Schoenhals J. E. Venkatesulu B. P. Anderson C. C. Barsoumian H. B. Younes A. I. (2018). Radiation therapy and immunotherapy: what is the optimal timing or sequencing? Immunotherapy 10, 299–316. 10.2217/IMT-2017-0082 29421979 PMC5810851

[B5] Allard B. Pommey S. Smyth M. J. Stagg J. (2013). Targeting CD73 enhances the antitumor activity of anti-PD-1 and anti-CTLA-4 mAbs. Clin. Cancer Res. 19, 5626–5635. 10.1158/1078-0432.CCR-13-0545 23983257

[B6] Allard D. Cousineau I. Ma E. H. Allard B. Bareche Y. Fleury H. (2023). The CD73 immune checkpoint promotes tumor cell metabolic fitness. Elife 12, e84508. 10.7554/ELIFE.84508 37261423 PMC10259490

[B7] Anscher M. S. Arora S. Weinstock C. Amatya A. Bandaru P. Tang C. (2022). Association of radiation therapy with risk of adverse events in patients receiving immunotherapy: a pooled analysis of trials in the US food and drug administration database. JAMA Oncol. 8, 232–240. 10.1001/JAMAONCOL.2021.6439 34989781 PMC8739815

[B8] Antonia S. J. Villegas A. Daniel D. Vicente D. Murakami S. Hui R. (2017). Durvalumab after chemoradiotherapy in stage III non–small-cell lung cancer. New England J. Med. 377, 1919–1929. 10.1056/nejmoa1709937 28885881

[B9] Antonia S. J. Villegas A. Daniel D. Vicente D. Murakami S. Hui R. (2018). Overall survival with durvalumab after chemoradiotherapy in stage III NSCLC. New England J. Med. 379, 2342–2350. 10.1056/nejmoa1809697 30280658

[B10] Antonioli L. Blandizzi C. Malavasi F. Ferrari D. Haskó G. (2016). Anti-CD73 immunotherapy: a viable way to reprogram the tumor microenvironment. Oncoimmunology 5, e1216292. 10.1080/2162402X.2016.1216292 27757316 PMC5048762

[B11] Antonioli L. Pacher P. Vizi E. S. Haskó G. (2013). CD39 and CD73 in immunity and inflammation. Trends Mol. Med. 19, 355–367. 10.1016/j.molmed.2013.03.005 23601906 PMC3674206

[B12] Azzam E. I. Jay-Gerin J. P. Pain D. (2012). Ionizing radiation-induced metabolic oxidative stress and prolonged cell injury. Cancer Lett. 327, 48–60. 10.1016/j.canlet.2011.12.012 22182453 PMC3980444

[B13] Bach N. Winzer R. Tolosa E. Fiedler W. Brauneck F. (2023). The clinical significance of CD73 in cancer. Int. J. Mol. Sci. 24, 11759. 10.3390/IJMS241411759 37511518 PMC10380759

[B14] Barcellos-Hoff M. H. Park C. Wright E. G. (2005). Radiation and the microenvironment - tumorigenesis and therapy. Nat. Rev. Cancer 5, 867–875. 10.1038/nrc1735 16327765

[B15] Barlesi F. Cho B. C. Goldberg S. B. Yoh K. Zimmer Gelatti A. C. Mann H. (2024). PACIFIC-9: phase III trial of durvalumab + oleclumab or monalizumab in unresectable stage III non-small-cell lung cancer. Future Oncol. 20, 2137–2147. 10.1080/14796694.2024.2354160 39023287 PMC11508940

[B16] Barletta K. E. Ley K. Mehrad B. (2012). Regulation of neutrophil function by adenosine. Arterioscler. Thromb. Vasc. Biol. 32, 856–864. 10.1161/ATVBAHA.111.226845 22423037 PMC3353547

[B17] Bauer F. N. Tertel T. Stambouli O. Wang C. Dittrich R. Staubach S. (2023). CD73 activity of mesenchymal stromal cell-derived extracellular vesicle preparations is detergent-resistant and does not correlate with immunomodulatory capabilities. Cytotherapy 25, 138–147. 10.1016/j.jcyt.2022.09.006 36244910

[B18] Beavis P. A. Divisekera U. Paget C. Chow M. T. John L. B. Devaud C. (2013). Blockade of A2A receptors potently suppresses the metastasis of CD73+ tumors. Proc. Natl. Acad. Sci. U. S. A. 110, 14711–14716. 10.1073/pnas.1308209110 23964122 PMC3767556

[B19] Beavis P. A. Milenkovski N. Henderson M. A. John L. B. Allard B. Loi S. (2015). Adenosine receptor 2A blockade increases the efficacy of anti-PD-1 through enhanced antitumor T-cell responses. Cancer Immunol. Res. 3, 506–517. 10.1158/2326-6066.CIR-14-0211 25672397

[B20] Bekker R. A. Kim S. Pilon-Thomas S. Enderling H. (2022). Mathematical modeling of radiotherapy and its impact on tumor interactions with the immune system. Neoplasia 28, 100796. 10.1016/J.NEO.2022.100796 35447601 PMC9043662

[B21] Belikoff B. G. Hatfield S. Georgiev P. Ohta A. Lukashev D. Buras J. A. (2011). A2B adenosine receptor blockade enhances macrophage-mediated bacterial phagocytosis and improves polymicrobial sepsis survival in mice. J. Immunol. 186, 2444–2453. 10.4049/JIMMUNOL.1001567 21242513 PMC3708265

[B22] Belka C. Ottinger H. Kreuzfelder E. Weinmann M. Lindemann M. Lepple-Wienhues A. (1999). Impact of localized radiotherapy on blood immune cells counts and function in humans. Radiotherapy Oncol. 50, 199–204. 10.1016/S0167-8140(98)00130-3 10368044

[B23] Bendell J. LoRusso P. Overman M. Noonan A. M. Kim D. W. Strickler J. H. (2023). First-in-human study of oleclumab, a potent, selective anti-CD73 monoclonal antibody, alone or in combination with durvalumab in patients with advanced solid tumors. Cancer Immunol. Immunother. 72, 2443–2458. 10.1007/s00262-023-03430-6 37016126 PMC10264501

[B24] Bissonnette E. Y. Lauzon-Joset J. F. Debley J. S. Ziegler S. F. (2020). Cross-talk between alveolar macrophages and lung epithelial cells is essential to maintain lung homeostasis. Front. Immunol. 11, 583042. 10.3389/fimmu.2020.583042 33178214 PMC7593577

[B25] Blanco-Melo D. Nilsson-Payant B. E. Liu W. C. Uhl S. Hoagland D. Møller R. (2020). Imbalanced host response to SARS-CoV-2 drives development of COVID-19. Cell 181, 1036–1045. 10.1016/J.CELL.2020.04.026 32416070 PMC7227586

[B26] Bledsoe T. J. Nath S. K. Decker R. H. (2017). Radiation pneumonitis. Clin. Chest Med. 38, 201–208. 10.1016/j.ccm.2016.12.004 28477633

[B27] Boerma M. Davis C. M. Jackson I. L. Schaue D. Williams J. P. (2022). All for one, though not one for all: team players in normal tissue radiobiology. Int. J. Radiat. Biol. 98, 346–366. 10.1080/09553002.2021.1941383 34129427 PMC8781287

[B28] Bönner F. Borg N. Burghoff S. Schrader J. (2012). Resident cardiac immune cells and expression of the ectonucleotidase enzymes CD39 and CD73 after ischemic injury. PLoS One 7, e34730. 10.1371/journal.pone.0034730 22514659 PMC3326036

[B29] Bopp T. Becker C. Klein M. Klein-Heßling S. Palmetshofer A. Serfling E. (2007). Cyclic adenosine monophosphate is a key component of regulatory T cell-mediated suppression. J. Exp. Med. 204, 1303–1310. 10.1084/JEM.20062129 17502663 PMC2118605

[B30] Boveda-Ruiz D. D’Alessandro-Gabazza C. N. Toda M. Takagi T. Naito M. Matsushima Y. (2013). Differential role of regulatory T cells in early and late stages of pulmonary fibrosis. Immunobiology 218, 245–254. 10.1016/j.imbio.2012.05.020 22739236

[B31] Buenestado A. Delyle S. G. Arnould I. Besnard F. Naline E. Blouquit-Laye S. (2010). The role of adenosine receptors in regulating production of tumour necrosis factor-alpha and chemokines by human lung macrophages. Br. J. Pharmacol. 159, 1304–1311. 10.1111/J.1476-5381.2009.00614.X 20136829 PMC2848934

[B32] Cao X. Zhu Z. Cao Y. Hu J. Min M. (2021). CD73 is a hypoxia-responsive gene and promotes the Warburg effect of human gastric cancer cells dependent on its enzyme activity. J. Cancer 12, 6372–6382. 10.7150/jca.62387 34659527 PMC8489133

[B33] Cappuccini F. Eldh T. Bruder D. Gereke M. Jastrow H. Schulze-Osthoff K. (2011). New insights into the molecular pathology of radiation-induced pneumopathy. Radiotherapy Oncol. 101, 86–92. 10.1016/j.radonc.2011.05.064 21722981

[B34] Cekic C. Linden J. (2014). Adenosine A2A receptors intrinsically regulate CD8+ T cells in the tumor microenvironment. Cancer Res. 74, 7239–7249. 10.1158/0008-5472.CAN-13-3581 25341542 PMC4459794

[B35] Cekic C. Linden J. (2016). Purinergic regulation of the immune system. Nat. Rev. Immunol. 16, 177–192. 10.1038/nri.2016.4 26922909

[B36] Cekic C. Sag D. Day Y. J. Linden J. (2013). Extracellular adenosine regulates naive T cell development and peripheral maintenance. J. Exp. Med. 210, 2693–2706. 10.1084/JEM.20130249 24145516 PMC3832923

[B37] Chambers A. M. Matosevic S. (2019). Immunometabolic dysfunction of natural killer cells mediated by the hypoxia-CD73 Axis in solid tumors. Front. Mol. Biosci. 6, 60. 10.3389/fmolb.2019.00060 31396523 PMC6668567

[B38] Chambers A. M. Wang J. Lupo K. B. Yu H. Atallah Lanman N. M. Matosevic S. (2018). Adenosinergic signaling alters natural killer cell functional responses. Front. Immunol. 9, 2533. 10.3389/fimmu.2018.02533 30425720 PMC6218627

[B39] Chatterjee D. Tufa D. M. Baehre H. Hass R. Schmidt R. E. Jacobs R. (2014). Natural killer cells acquire CD73 expression upon exposure to mesenchymal stem cells. Blood 123, 594–595. 10.1182/BLOOD-2013-09-524827 24458278

[B40] Chen F. Niu J. Wang M. Zhu H. Guo Z. (2023). Re-evaluating the risk factors for radiation pneumonitis in the era of immunotherapy. J. Transl. Med. 21, 368. 10.1186/s12967-023-04212-5 37287014 PMC10246421

[B41] Chen H. Yang D. Carroll S. H. Eltzschig H. K. Ravid K. (2009). Activation of the macrophage A2b adenosine receptor regulates tumor necrosis factor-alpha levels following vascular injury. Exp. Hematol. 37, 533–538. 10.1016/J.EXPHEM.2009.02.001 19375644 PMC5748295

[B42] Chen X. Sheikh K. Nakajima E. Lin C. T. Lee J. Hu C. (2021). Radiation versus immune checkpoint inhibitor associated pneumonitis: distinct radiologic morphologies. Oncologist 26, e1822–e1832. 10.1002/ONCO.13900 34251728 PMC8488797

[B43] Citrin D. E. Shankavaram U. Horton J. A. Shield W. Zhao S. Asano H. (2013). Role of type II pneumocyte senescence in radiation-induced lung fibrosis. J. Natl. Cancer Inst. 105, 1474–1484. 10.1093/JNCI/DJT212 24052614 PMC3787909

[B44] Clemans D. L. Bauer R. J. Hanson J. A. Hobbs M. V. St. Geme J. W. Marrs C. F. (2000). Induction of proinflammatory cytokines from human respiratory epithelial cells after stimulation by nontypeable Haemophilus influenzae. Infect. Immun. 68, 4430–4440. 10.1128/IAI.68.8.4430-4440.2000 10899840 PMC98342

[B45] Colgan S. P. Eltzschig H. K. Eckle T. Thompson L. F. (2006). Physiological roles for ecto-5′-nucleotidase (CD73). Purinergic Signal 2, 351–360. 10.1007/s11302-005-5302-5 18404475 PMC2254482

[B46] Cook P. C. MacDonald A. S. (2016). Dendritic cells in lung immunopathology. Seminars Immunopathol. 2016 38, 449–460. 10.1007/S00281-016-0571-3 PMC4896986 27256370

[B47] Cortiula F. Reymen B. Peters S. Van Mol P. Wauters E. Vansteenkiste J. (2022). Immunotherapy in unresectable stage III non-small-cell lung cancer: state of the art and novel therapeutic approaches. Ann. Oncol. 33, 893–908. 10.1016/j.annonc.2022.06.013 35777706

[B48] Cronstein B. N. Daguma L. Nichols D. Hutchison A. J. Williams M. (1990). The adenosine/neutrophil paradox resolved: human neutrophils possess both A1 and A2 receptors that promote chemotaxis and inhibit O2 generation, respectively. J. Clin. Invest 85, 1150–1157. 10.1172/JCI114547 2156895 PMC296546

[B49] Cronstein B. N. Rosenstein E. D. Kramer S. B. Weissmann G. Hirschhorn R. (1985). Adenosine; a physiologic modulator of superoxide anion generation by human neutrophils. Adenosine acts via an A2 receptor on human neutrophils. J. Immunol. 135, 1366–1371. 10.4049/jimmunol.135.2.1366 2989364

[B50] Csóka B. Himer L. Selmeczy Z. Vizi E. S. Pacher P. Ledent C. (2008). Adenosine A2A receptor activation inhibits T helper 1 and T helper 2 cell development and effector function. FASEB J. 22, 3491–3499. 10.1096/FJ.08-107458 18625677 PMC2537430

[B51] Cunliffe A. Armato S. G. Castillo R. Pham N. Guerrero T. Al-Hallaq H. A. (2015). Lung texture in serial thoracic computed tomography scans: correlation of radiomics-based features with radiation therapy dose and radiation pneumonitis development. Int. J. Radiat. Oncol. Biol. Phys. 91, 1048–1056. 10.1016/j.ijrobp.2014.11.030 25670540 PMC4383676

[B52] Cytlak U. M. Dyer D. P. Honeychurch J. Williams K. J. Travis M. A. Illidge T. M. (2022). Immunomodulation by radiotherapy in tumour control and normal tissue toxicity. Nat. Rev. Immunol. 22, 124–138. 10.1038/s41577-021-00568-1 34211187

[B53] Deaglio S. Dwyer K. M. Gao W. Friedman D. Usheva A. Erat A. (2007). Adenosine generation catalyzed by CD39 and CD73 expressed on regulatory T cells mediates immune suppression. J. Exp. Med. 204, 1257–1265. 10.1084/JEM.20062512 17502665 PMC2118603

[B54] Deaglio S. Robson S. C. (2011). Ectonucleotidases as regulators of purinergic signaling in thrombosis, inflammation, and immunity. Adv. Pharmacol. 61, 301–332. 10.1016/B978-0-12-385526-8.00010-2 21586363 PMC5879773

[B55] De Leve S. Wirsdörfer F. Cappuccini F. Schütze A. Meyer A. V. Röck K. (2017). Loss of CD73 prevents accumulation of alternatively activated macrophages and the formation of prefibrotic macrophage clusters in irradiated lungs. FASEB J. 31, 2869–2880. 10.1096/fj.201601228R 28325757 PMC6137497

[B56] De Leve S. Wirsdörfer F. Jendrossek V. (2019a). Targeting the immunomodulatory CD73/adenosine system to improve the therapeutic gain of radiotherapy. Front. Immunol. 10, 698. 10.3389/fimmu.2019.00698 31024543 PMC6460721

[B57] De Leve S. Wirsdörfer F. Jendrossek V. (2019b). The CD73/ado system—a new player in RT induced adverse late effects. Cancers 11, 1578. 10.3390/CANCERS11101578 31623231 PMC6827091

[B58] Denekamp J. Rojas A. (1989). Cell kinetics and radiation pathology. Experientia 45, 33–41. 10.1007/BF01990450 2643525

[B59] Dominici M. Le Blanc K. Mueller I. Slaper-Cortenbach I. Marini F. C. Krause D. S. (2006). Minimal criteria for defining multipotent mesenchymal stromal cells. The International Society for Cellular Therapy position statement. Cytotherapy 8, 315–317. 10.1080/14653240600855905 16923606

[B60] Dwyer K. M. Deaglio S. Gao W. Friedman D. Strom T. B. Robson S. C. (2007). CD39 and control of cellular immune responses. Purinergic Signal 3, 171–180. 10.1007/S11302-006-9050-Y 18404431 PMC2096766

[B61] Eckle T. Füllbier L. Wehrmann M. Khoury J. Mittelbronn M. Ibla J. (2007). Identification of ectonucleotidases CD39 and CD73 in innate protection during acute lung injury. J. Immunol. 178, 8127–8137. 10.4049/JIMMUNOL.178.12.8127 17548651

[B62] Ehrentraut H. Westrich J. A. Eltzschig H. K. Clambey E. T. (2012). Adora2b adenosine receptor engagement enhances regulatory T cell abundance during endotoxin-induced pulmonary inflammation. PLoS One 7, e32416. 10.1371/JOURNAL.PONE.0032416 22389701 PMC3289657

[B63] Elliott M. R. Chekeni F. B. Trampont P. C. Lazarowski E. R. Kadl A. Walk S. F. (2009). Nucleotides released by apoptotic cells act as a find-me signal to promote phagocytic clearance. Nat. 2009 461, 282–286. 10.1038/nature08296 PMC2851546 19741708

[B64] Eltzschig H. K. MacManus C. F. Colgan S. P. (2008). Neutrophils as sources of extracellular nucleotides: functional consequences at the vascular interface. Trends Cardiovasc Med. 18, 103–107. 10.1016/J.TCM.2008.01.006 18436149 PMC2711033

[B65] Eltzschig H. K. Thompson L. F. Karhausen J. Cotta R. J. Ibla J. C. Robson S. C. (2004). Endogenous adenosine produced during hypoxia attenuates neutrophil accumulation: coordination by extracellular nucleotide metabolism. Blood 104, 3986–3992. 10.1182/BLOOD-2004-06-2066 15319286

[B66] Erdmann A. A. Gao Z. G. Jung U. Foley J. Borenstein T. Jacobson K. A. (2005). Activation of Th1 and Tc1 cell adenosine A2A receptors directly inhibits IL-2 secretion *in vitro* and IL-2-driven expansion *in vivo* . Blood 105, 4707–4714. 10.1182/BLOOD-2004-04-1407 15746085 PMC1895001

[B67] Ernens I. Léonard F. Vausort M. Rolland-Turner M. Devaux Y. Wagner D. R. (2010). Adenosine up-regulates vascular endothelial growth factor in human macrophages. Biochem. Biophys. Res. Commun. 392, 351–356. 10.1016/J.BBRC.2010.01.023 20067761

[B68] Factor P. Mutlu G. M. Chen L. Mohameed J. Akhmedov A. T. Fan J. M. (2007). Adenosine regulation of alveolar fluid clearance. Proc. Natl. Acad. Sci. U. S. A. 104, 4083–4088. 10.1073/pnas.0601117104 17360481 PMC1820712

[B69] Faehling M. Schumann C. Christopoulos P. Hoffknecht P. Alt J. Horn M. (2020). Durvalumab after definitive chemoradiotherapy in locally advanced unresectable non-small cell lung cancer (NSCLC): real-world data on survival and safety from the German expanded-access program (EAP). Lung Cancer 150, 114–122. 10.1016/j.lungcan.2020.10.006 33126091

[B70] Faivre-Finn C. Vicente D. Kurata T. Planchard D. Paz-Ares L. Vansteenkiste J. F. (2021). Four-year survival with durvalumab after chemoradiotherapy in stage III NSCLC—an update from the PACIFIC trial. J. Thorac. Oncol. 16, 860–867. 10.1016/j.jtho.2020.12.015 33476803

[B71] Ferretti E. Horenstein A. L. Canzonetta C. Costa F. Morandi F. (2019). Canonical and non-canonical adenosinergic pathways. Immunol. Lett. 205, 25–30. 10.1016/j.imlet.2018.03.007 29550257

[B72] Folkesson H. G. Kuzenko S. R. Lipson D. A. Matthay M. A. Simmons M. A. (2012). The adenosine 2A receptor agonist GW328267C improves lung function after acute lung injury in rats. Am. J. Physiol. Lung Cell Mol. Physiol. 303, 259–271. 10.1152/ajplung.00395.2011 22659881

[B73] Gagliostro V. Seeger P. Garrafa E. Salvi V. Bresciani R. Bosisio D. (2016). Pro-lymphangiogenic properties of IFN-γ-activated human dendritic cells. Immunol. Lett. 173, 26–35. 10.1016/J.IMLET.2016.03.008 26987844

[B74] Geng Y. Su S. Cao L. Yang T. Ouyang W. Liu L. (2022). Effect of PD-1 inhibitor combined with X-ray irradiation on the inflammatory microenvironment and lung tissue injury in mice. J. Inflamm. Res. 15, 545–556. 10.2147/JIR.S350112 35115804 PMC8803086

[B75] Girard N. Bar J. Garrido P. Garassino M. C. McDonald F. Mornex F. (2023). Treatment characteristics and real-world progression-free survival in patients with unresectable stage III NSCLC who received durvalumab after chemoradiotherapy: findings from the PACIFIC-R study. J. Thorac. Oncol. 18, 181–193. 10.1016/j.jtho.2022.10.003 36307040

[B76] Giuliani P. Carluccio M. Ciccarelli R. (2021). Role of purinome, A complex signaling system, in glioblastoma aggressiveness. Front. Pharmacol. 12, 632622. 10.3389/fphar.2021.632622 33613296 PMC7892952

[B77] Giuranno L. Ient J. De Ruysscher D. Vooijs M. A. (2019). Radiation-induced lung injury (RILI). Front. Oncol. 9, 877. 10.3389/fonc.2019.00877 31555602 PMC6743286

[B78] Glaser L. Coulter P. J. Shields M. Touzelet O. Power U. F. Broadbent L. (2019). Airway epithelial derived cytokines and chemokines and their role in the immune response to respiratory syncytial virus infection. Pathogens 8, 106. 10.3390/PATHOGENS8030106 31331089 PMC6789711

[B79] Golden E. B. Frances D. Pellicciotta I. Demaria S. Barcellos-Hoff M. H. Formenti S. C. (2014). Radiation fosters dose-dependent and chemotherapy-induced immunogenic cell death. Oncoimmunology 3, e28518. 10.4161/ONCI.28518 25071979 PMC4106151

[B80] Gomez G. Sitkovsky M. V. (2003). Differential requirement for A2a and A3 adenosine receptors for the protective effect of inosine *in vivo* . Blood 102, 4472–4478. 10.1182/BLOOD-2002-11-3624 12947007

[B81] Gottschling S. Granzow M. Kuner R. Jauch A. Herpel E. Xu E. C. (2013). Mesenchymal stem cells in non-small cell lung cancer-Different from others? Insights from comparative molecular and functional analyses. Lung Cancer 80, 19–29. 10.1016/j.lungcan.2012.12.015 23294501

[B82] Gowhari Shabgah A. Haleem Al-qaim Z. Markov A. Valerievich Yumashev A. Ezzatifar F. Ahmadi M. (2021). Chemokine CXCL14; a double-edged sword in cancer development. Int. Immunopharmacol. 97, 107681. 10.1016/J.INTIMP.2021.107681 33932697

[B83] Guberina N. Wirsdörfer F. Stuschke M. Jendrossek V. (2023). Combined radiation- and immune checkpoint-inhibitor-induced pneumonitis – the challenge to predict and detect overlapping immune-related adverse effects from evolving laboratory biomarkers and clinical imaging. Neoplasia (United States) 39, 100892. 10.1016/j.neo.2023.100892 PMC10124136 37011458

[B84] Guo T. Zou L. Ni J. Zhou Y. Ye L. Yang X. (2020). Regulatory T cells: an emerging player in radiation-induced lung injury. Front. Immunol. 11, 1769–9. 10.3389/fimmu.2020.01769 32849634 PMC7417370

[B85] Gupta K. Perkerson R. B. Parsons T. M. Angom R. Amerna D. Burgess J. D. (2024). Secretome from iPSC-derived MSCs exerts proangiogenic and immunosuppressive effects to alleviate radiation-induced vascular endothelial cell damage. Stem Cell Res. Ther. 15, 230. 10.1186/s13287-024-03847-5 39075600 PMC11287895

[B86] Han Y. Lee T. He Y. Raman R. Irizarry A. Martin M. L. (2022). The regulation of CD73 in non-small cell lung cancer. Eur. J. Cancer 170, 91–102. 10.1016/J.EJCA.2022.04.025 35598361

[B87] Hansel C. Jendrossek V. Klein D. (2020). Cellular senescence in the lung: the central role of senescent epithelial cells. Int. J. Mol. Sci. 21, 3279. 10.3390/ijms21093279 32384619 PMC7247355

[B88] Haskó G. Cronstein B. N. (2004). Adenosine: an endogenous regulator of innate immunity. Trends Immunol. 25, 33–39. 10.1016/J.IT.2003.11.003 14698282

[B89] Hatfield S. M. Kjaergaard J. Lukashev D. Schreiber T. H. Belikoff B. Abbott R. (2015). Immunological mechanisms of the antitumor effects of supplemental oxygenation. Sci. Transl. Med. 7, 277ra30. 10.1126/scitranslmed.aaa1260 PMC4641038 25739764

[B90] He X. Xu C. (2020). Immune checkpoint signaling and cancer immunotherapy. Cell Res. 2020 30, 660–669. 10.1038/s41422-020-0343-4 PMC7395714 32467592

[B91] Herbst R. S. Majem M. Barlesi F. Carcereny E. Chu Q. Monnet I. (2022). COAST: an open-label, phase II, multidrug platform study of durvalumab alone or in combination with oleclumab or monalizumab in patients with unresectable, stage III non-small-cell lung cancer. J. Clin. Oncol. 40, 3383–3393. 10.1200/JCO.22.00227 35452273

[B92] Heylmann D. Rödel F. Kindler T. Kaina B. (2014). Radiation sensitivity of human and murine peripheral blood lymphocytes, stem and progenitor cells. Biochim. Biophys. Acta 1846, 121–129. 10.1016/J.BBCAN.2014.04.009 24797212

[B93] Hoegl S. Brodsky K. S. Blackburn M. R. Karmouty-Quintana H. Zwissler B. Eltzschig H. K. (2015). Alveolar epithelial A2B adenosine receptors in pulmonary protection during acute lung injury. J. Immunol. 195, 1815–1824. 10.4049/jimmunol.1401957 26188061 PMC4530072

[B94] Hope A. Verduin M. Dilling T. J. Choudhury A. Fijten R. Wee L. (2021). Artificial intelligence applications to improve the treatment of locally advanced non-small cell lung cancers. Cancers (Basel) 13, 2382. 10.3390/cancers13102382 34069307 PMC8156328

[B95] Hou G. Li J. Liu W. Wei J. Xin Y. Jiang X. (2022). Mesenchymal stem cells in radiation-induced lung injury: from mechanisms to therapeutic potential. Front. Cell Dev. Biol. 10, 1100305. 10.3389/fcell.2022.1100305 36578783 PMC9790971

[B96] Huang Z. Xie N. Illes P. Di Virgilio F. Ulrich H. Semyanov A. (2021). From purines to purinergic signalling: molecular functions and human diseases. Signal Transduct. Target Ther. 6, 162. 10.1038/s41392-021-00553-z 33907179 PMC8079716

[B97] Iannone R. Miele L. Maiolino P. Pinto A. Morello S. (2013). Blockade of A2b adenosine receptor reduces tumor growth and immune suppression mediated by myeloid-derived suppressor cells in a mouse model of melanoma. Neoplasia 15, 1400–1409. 10.1593/NEO.131748 24403862 PMC3884531

[B98] Inoue Y. Yoshimura K. Kurabe N. Kahyo T. Kawase A. Tanahashi M. (2017). Prognostic impact of CD73 and A2A adenosine receptor expression in non-small-cell lung cancer. Oncotarget 8, 8738–8751. 10.18632/oncotarget.14434 28060732 PMC5352437

[B99] Jarzebska N. Karetnikova E. S. Markov A. G. Kasper M. Rodionov R. N. Spieth P. M. (2021). Scarred lung. An update on radiation-induced pulmonary fibrosis. Front. Med. (Lausanne) 7, 585756. 10.3389/fmed.2020.585756 33521012 PMC7843914

[B100] Jeske S. S. Brand M. Ziebart A. Laban S. Doescher J. Greve J. (2020). Adenosine-producing regulatory B cells in head and neck cancer. Cancer Immunol. Immunother. 69, 1205–1216. 10.1007/s00262-020-02535-6 32146518 PMC7303082

[B101] Jung H. A. Noh J. M. Sun J. M. Lee S. H. Ahn J. S. Ahn M. J. (2020). Real world data of durvalumab consolidation after chemoradiotherapy in stage III non-small-cell lung cancer. Lung Cancer 146, 23–29. 10.1016/j.lungcan.2020.05.035 32505077

[B102] Käsmann L. Dietrich A. Staab-Weijnitz C. A. Manapov F. Behr J. Rimner A. (2020). Radiation-induced lung toxicity - cellular and molecular mechanisms of pathogenesis, management, and literature review. Radiat. Oncol. 15, 214. 10.1186/S13014-020-01654-9 32912295 PMC7488099

[B103] Kaur J. Dora S. (2023). Purinergic signaling: diverse effects and therapeutic potential in cancer. Front. Oncol. 13, 1058371. 10.3389/fonc.2023.1058371 36741002 PMC9889871

[B104] Kinsey G. R. Huang L. Jaworska K. Khutsishvili K. Becker D. A. Ye H. (2012). Autocrine adenosine signaling promotes regulatory T cell-mediated renal protection. J. Am. Soc. Nephrol. 23, 1528–1537. 10.1681/ASN.2012010070 22835488 PMC3431416

[B105] Kjaergaard J. Hatfield S. Jones G. Ohta A. Sitkovsky M. (2018). A2A adenosine receptor gene deletion or synthetic A2A antagonist liberate tumor-reactive CD8+ T cells from tumor-induced immunosuppression. J. Immunol. 201, 782–791. 10.4049/jimmunol.1700850 29802128 PMC6052792

[B106] Klein D. Schmetter A. Imsak R. Wirsdörfer F. Unger K. Jastrow H. (2016). Therapy with multipotent mesenchymal stromal cells protects lungs from radiation-induced injury and reduces the risk of lung metastasis. Antioxid. Redox Signal 24, 53–69. 10.1089/ARS.2014.6183 26066676

[B107] Klein D. Steens J. Wiesemann A. Schulz F. Kaschani F. Röck K. (2017). Mesenchymal stem cell therapy protects lungs from radiation-induced endothelial cell loss by restoring superoxide dismutase 1 expression. Antioxid. Redox Signal 26, 563–582. 10.1089/ars.2016.6748 27572073 PMC5393411

[B108] Koszalka P. Özüyaman B. Huo Y. Zernecke A. Flögel U. Braun N. (2004). Targeted disruption of cd73/ecto-5′-nucleotidase alters thromboregulation and augments vascular inflammatory response. Circ. Res. 95, 814–821. 10.1161/01.RES.0000144796.82787.6F 15358667

[B109] Kou M. Huang L. Yang J. Chiang Z. Chen S. Liu J. (2022). Mesenchymal stem cell-derived extracellular vesicles for immunomodulation and regeneration: a next generation therapeutic tool? Cell Death Dis. 13, 580. 10.1038/s41419-022-05034-x 35787632 PMC9252569

[B110] Kowash R. R. Akbay E. A. (2023). Tumor intrinsic and extrinsic functions of CD73 and the adenosine pathway in lung cancer. Front. Immunol. 14, 1130358. 10.3389/FIMMU.2023.1130358 37033953 PMC10079876

[B111] Krafft S. P. Rao A. Stingo F. Briere T. M. Court L. E. Liao Z. (2018). The utility of quantitative CT radiomics features for improved prediction of radiation pneumonitis. Med. Phys. 45, 5317–5324. 10.1002/mp.13150 30133809

[B112] Kreckler L. M. Wan T. C. Ge Z. D. Auchampach J. A. (2006). Adenosine inhibits tumor necrosis factor-alpha release from mouse peritoneal macrophages via A2A and A2B but not the A3 adenosine receptor. J. Pharmacol. Exp. Ther. 317, 172–180. 10.1124/JPET.105.096016 16339914

[B113] Kuang Y. Pierce C. M. Chang H. C. Sosinsky A. Z. Deitz A. C. Keller S. M. (2022). Chemoradiation-induced pneumonitis in patients with unresectable stage III non-small cell lung cancer: a systematic literature review and meta-analysis. Lung Cancer 174, 174–185. 10.1016/j.lungcan.2022.06.005 35717343

[B114] Land W. G. (2015). The role of damage-associated molecular patterns (DAMPs) in human diseases: Part II: DAMPs as diagnostics, prognostics and therapeutics in clinical medicine. Sultan Qaboos Univ. Med. J. 15, e157–e170. 26052447 PMC4450777

[B115] Lappas C. M. Rieger J. M. Linden J. (2005). A2A adenosine receptor induction inhibits IFN-gamma production in murine CD4+ T cells. J. Immunol. 174, 1073–1080. 10.4049/JIMMUNOL.174.2.1073 15634932

[B116] Lazarowski E. R. Mason S. J. Clarke L. Harden T. K. Boucher R. C. (1992). Adenosine receptors on human airway epithelia and their relationship to chloride secretion. Br. J. Pharmacol. 106, 774–782. 10.1111/J.1476-5381.1992.TB14412.X 1327386 PMC1907665

[B117] Leavy O. (2013). T cells: adenosine maintains the numbers. Nat. Rev. Immunol. 13, 848–849. 10.1038/nri3571 24232464

[B118] Lee J. Park H. Moon S. Do J. T. Hong K. Choi Y. (2021). Expression and regulation of CD73 during the estrous cycle in mouse uterus. Int. J. Mol. Sci. 22, 9403. 10.3390/ijms22179403 34502315 PMC8431015

[B119] Lee Y. H. Choi H. S. Jeong H. Kang K. M. Song J. H. Lee W. S. (2018). Neutrophil-lymphocyte ratio and a dosimetric factor for predicting symptomatic radiation pneumonitis in non-small-cell lung cancer patients treated with concurrent chemoradiotherapy. Clin. Respir. J. 12, 1264–1273. 10.1111/CRJ.12660 28618180

[B120] Lei X. He N. Zhu L. Zhou M. Zhang K. Wang C. (2021). Mesenchymal stem cell-derived extracellular vesicles attenuate radiation-induced lung injury via miRNA-214-3p. Antioxid. Redox Signal 35, 849–862. 10.1089/ars.2019.7965 32664737

[B121] Leone R. D. Sun I. M. Oh M. H. Sun I. H. Wen J. Englert J. (2018). Inhibition of the adenosine A2a receptor modulates expression of T cell coinhibitory receptors and improves effector function for enhanced checkpoint blockade and ACT in murine cancer models. Cancer Immunol. Immunother. 67, 1271–1284. 10.1007/s00262-018-2186-0 29923026 PMC11028354

[B122] Lhuillier C. Rudqvist N. P. Elemento O. Formenti S. C. Demaria S. (2019). Radiation therapy and anti-tumor immunity: exposing immunogenic mutations to the immune system. Genome Med. 11, 40. 10.1186/s13073-019-0653-7 31221199 PMC6587285

[B123] Li Y. Shen Z. Jiang X. Wang Y. Yang Z. Mao Y. (2022). Mouse mesenchymal stem cell-derived exosomal miR-466f-3p reverses EMT process through inhibiting AKT/GSK3β pathway via c-MET in radiation-induced lung injury. J. Exp. Clin. Cancer Res. 41, 128. 10.1186/s13046-022-02351-z 35392967 PMC8988379

[B124] Lierova A. Jelicova M. Nemcova M. Proksova M. Pejchal J. Zarybnicka L. (2018). Cytokines and radiation-induced pulmonary injuries. J. Radiat. Res. 59, 709–753. 10.1093/jrr/rry067 30169853 PMC6251431

[B125] Lin X. Deng H. Yang Y. Wu J. Qiu G. Li S. (2021). Peripheral blood biomarkers for early diagnosis, severity, and prognosis of checkpoint inhibitor-related pneumonitis in patients with lung cancer. Front. Oncol. 11, 698832. 10.3389/fonc.2021.698832 34327140 PMC8313853

[B126] Lin Y. S. Chiang S. F. Chen C. Y. Hong W. Z. Chen T. W. Chen W. T. L. (2023). Targeting CD73 increases therapeutic response to immunogenic chemotherapy by promoting dendritic cell maturation. Cancer Immunol. Immunother. 72, 2283–2297. 10.1007/s00262-023-03416-4 36881132 PMC10991491

[B127] Linden J. (2006). Cell biology. Purinergic chemotaxis. Sci. (1979) 314, 1689–1690. 10.1126/SCIENCE.1137190 17170280

[B128] Liu B. Wang Y. Han G. Zhu M. (2023). Tolerogenic dendritic cells in radiation-induced lung injury. Front. Immunol. 14, 1323676. 10.3389/fimmu.2023.1323676 38259434 PMC10800505

[B129] Ma X. L. Shen M. N. Hu B. Wang B. L. Yang W. J. Lv L. H. (2019). CD73 promotes hepatocellular carcinoma progression and metastasis via activating PI3K/AKT signaling by inducing Rap1-mediated membrane localization of P110β and predicts poor prognosis. J. Hematol. Oncol. 12, 37. 10.1186/s13045-019-0724-7 30971294 PMC6458749

[B130] Mandapathil M. Hilldorfer B. Szczepanski M. J. Czystowska M. Szajnik M. Ren J. (2010). Generation and accumulation of immunosuppressive adenosine by human CD4+CD25highFOXP3+ regulatory T Cells. J. Biol. Chem. 285, 7176–7186. 10.1074/JBC.M109.047423 19858205 PMC2844167

[B131] Mastelic-Gavillet B. Navarro Rodrigo B. Décombaz L. Wang H. Ercolano G. Ahmed R. (2019). Adenosine mediates functional and metabolic suppression of peripheral and tumor-infiltrating CD8+ T cells. J. Immunother. Cancer 7, 257. 10.1186/s40425-019-0719-5 31601268 PMC6788118

[B132] McColl S. R. St-Onge M. Dussault A.-A. Laflamme C. Bouchard L. Boulanger J. (2006). Immunomodulatory impact of the A2A adenosine receptor on the profile of chemokines produced by neutrophils. FASEB J. 20, 187–189. 10.1096/FJ.05-4804FJE 16280366 PMC2881301

[B133] Merad M. Sathe P. Helft J. Miller J. Mortha A. (2013). The dendritic cell lineage: ontogeny and function of dendritic cells and their subsets in the steady state and the inflamed setting. Annu. Rev. Immunol. 31, 563–604. 10.1146/ANNUREV-IMMUNOL-020711-074950 23516985 PMC3853342

[B134] Meyer A. V. Klein D. de Leve S. Szymonowicz K. Stuschke M. Robson S. C. (2020). Host CD39 deficiency affects radiation-induced tumor growth delay and aggravates radiation-induced normal tissue toxicity. Front. Oncol. 10, 554883. 10.3389/fonc.2020.554883 33194619 PMC7649817

[B135] Mohamed S. Bertolaccini L. Galetta D. Petrella F. Casiraghi M. de Marinis F. (2023). The role of immunotherapy or immuno-chemotherapy in non-small cell lung cancer: a comprehensive review. Cancers 2023 15, 2476. 10.3390/CANCERS15092476 PMC10177497 37173943

[B136] Montay-Gruel P. Zhu Y. Petit B. Leavitt R. Warn M. Giedzinski E. (2021). Extracellular vesicles for the treatment of radiation-induced normal tissue toxicity in the lung. Front. Oncol. 10, 602763. 10.3389/fonc.2020.602763 33738245 PMC7962869

[B137] Moratiel-Pellitero A. Zapata-García M. Gascón-Ruiz M. Sesma A. Quílez E. Ramirez-Labrada A. (2024). Biomarkers of immunotherapy response in patients with non-small-cell lung cancer: microbiota composition, short-chain fatty acids, and intestinal permeability. Cancers 16, 1144. 10.3390/CANCERS16061144 38539479 PMC10969216

[B138] Murao A. Aziz M. Wang H. Brenner M. Wang P. (2021). Release mechanisms of major DAMPs. Apoptosis 26, 152–162. 10.1007/s10495-021-01663-3 33713214 PMC8016797

[B139] Naidoo J. Nishino M. Patel S. P. Shankar B. Rekhtman N. Illei P. (2020). Immune-related pneumonitis after chemoradiotherapy and subsequent immune checkpoint blockade in unresectable stage III non–small-cell lung cancer. Clin. Lung Cancer 21, e435–e444. 10.1016/j.cllc.2020.02.025 32576443

[B140] Nakatsukasa H. Tsukimoto M. Harada H. Kojima S. (2011). Adenosine A2B receptor antagonist suppresses differentiation to regulatory T cells without suppressing activation of T cells. Biochem. Biophys. Res. Commun. 409, 114–119. 10.1016/J.BBRC.2011.04.125 21557932

[B141] Ndzie Noah M. L. Adzika G. K. Mprah R. Adekunle A. O. Koda S. Adu-Amankwaah J. (2023). Estrogen downregulates CD73/adenosine axis hyperactivity via adaptive modulation PI3K/Akt signaling to prevent myocarditis and arrhythmias during chronic catecholamines stress. Cell Commun. Signal. 21, 41. 10.1186/s12964-023-01052-0 36823590 PMC9948346

[B142] Németh Z. H. Lutz C. S. Csóka B. Deitch E. A. Leibovich S. J. Gause W. C. (2005). Adenosine augments IL-10 production by macrophages through an A2B receptor-mediated posttranscriptional mechanism. J. Immunol. 175, 8260–8270. 10.4049/JIMMUNOL.175.12.8260 16339566 PMC2000336

[B143] Neo S. Y. Yang Y. Record J. Ma R. Chen X. Chen Z. (2020). CD73 immune checkpoint defines regulatory NK cells within the tumor microenvironment. J. Clin. Invest 130, 1185–1198. 10.1172/JCI128895 31770109 PMC7269592

[B144] Nikolova M. Carriere M. Jenabian M. A. Limou S. Younas M. Kök A. (2011). CD39/adenosine pathway is involved in AIDS progression. PLoS Pathog. 7, e1002110. 10.1371/journal.ppat.1002110 21750674 PMC3131268

[B145] Nishimura A. Ono A. Wakuda K. Kawabata T. Yabe M. Miyawaki T. (2022). Prognostic impact of pneumonitis after durvalumab therapy in patients with locally advanced non-small cell lung cancer. Invest New Drugs 40, 403–410. 10.1007/S10637-021-01191-6 34633575 PMC8993741

[B146] Novitskiy S. V. Ryzhov S. Zaynagetdinov R. Goldstein A. E. Huang Y. Tikhomirov O. Y. (2008). Adenosine receptors in regulation of dendritic cell differentiation and function. Blood 112, 1822–1831. 10.1182/BLOOD-2008-02-136325 18559975 PMC2518889

[B147] Ohta A. Kini R. Ohta A. Subramanian M. Madasu M. Sitkovsky M. (2012). The development and immunosuppressive functions of CD4(+) CD25(+) FoxP3(+) regulatory T cells are under influence of the adenosine-A2A adenosine receptor pathway. Front. Immunol. 3, 190. 10.3389/FIMMU.2012.00190 22783261 PMC3389649

[B148] Pacheco J. M. Schenk E. L. (2021). CD73 and adenosine receptor signaling as a potential therapeutic target in EGFR-mutated NSCLC. J. Thorac. Oncol. 16, 509–511. 10.1016/J.JTHO.2021.01.1623 33781438

[B149] Petrovic-Djergovic D. Hyman M. C. Ray J. J. Bouis D. Visovatti S. H. Hayasaki T. (2012). Tissue-resident ecto-5′ nucleotidase (CD73) regulates leukocyte trafficking in the ischemic brain. J. Immunol. 188, 2387–2398. 10.4049/JIMMUNOL.1003671 22291183 PMC3644975

[B150] Picher M. Burch L. H. Hirsh A. J. Spychala J. Boucher R. C. (2003). Ecto 5′-nucleotidase and nonspecific alkaline phosphatase: two amp-hydrolyzing ectoenzymes with distinct roles in human airways. J. Biol. Chem. 278, 13468–13479. 10.1074/JBC.M300569200 12560324

[B151] Pillai R. N. Behera M. Owonikoko T. K. Kamphorst A. O. Pakkala S. Belani C. P. (2018). Comparison of the toxicity profile of PD-1 versus PD-L1 inhibitors in non-small cell lung cancer: a systematic analysis of the literature. Cancer 124, 271–277. 10.1002/CNCR.31043 28960263 PMC5761314

[B152] Ponce N. E. Sanmarco L. M. Eberhardt N. García M. C. Rivarola H. W. Cano R. C. (2016). CD73 inhibition shifts cardiac macrophage polarization toward a microbicidal phenotype and ameliorates the outcome of experimental chagas cardiomyopathy. J. Immunol. 197, 814–823. 10.4049/JIMMUNOL.1600371 27335499

[B153] Przybyla T. Sakowicz-Burkiewicz M. Pawelczyk T. (2018). Purinergic signaling in B cells. Acta Biochim. Pol. 65, 1–7. 10.18388/ABP.2017_1588 29360885

[B154] Rahi M. S. Parekh J. Pednekar P. Parmar G. Abraham S. Nasir S. (2021). Radiation-induced lung injury—current perspectives and management. Clin. Pract. 11, 410–429. 10.3390/clinpract11030056 34287252 PMC8293129

[B155] Ramanathan M. Pinhal-Enfield G. Hao I. Leibovich S. J. (2007). Synergistic up-regulation of vascular endothelial growth factor (VEGF) expression in macrophages by adenosine A2A receptor agonists and endotoxin involves transcriptional regulation via the hypoxia response element in the VEGF promoter. Mol. Biol. Cell 18, 14–23. 10.1091/MBC.E06-07-0596 17065555 PMC1751314

[B156] Ramos T. L. Sánchez-Abarca L. I. Muntión S. Preciado S. Puig N. López-Ruano G. (2016). MSC surface markers (CD44, CD73, and CD90) can identify human MSC-derived extracellular vesicles by conventional flow cytometry. Cell Commun. Signal. 14, 2. 10.1186/s12964-015-0124-8 26754424 PMC4709865

[B157] Raskovalova T. Lokshin A. Huang X. Jackson E. K. Gorelik E. (2006). Adenosine-mediated inhibition of cytotoxic activity and cytokine production by IL-2/NKp46-activated NK cells: involvement of protein kinase A isozyme I (PKA I). Immunol. Res. 36, 91–99. 10.1385/IR:36:1:91 17337770

[B158] Remon J. Levy A. Singh P. Hendriks L. E. L. Aldea M. Arrieta O. (2022). Current challenges of unresectable stage III NSCLC: are we ready to break the glass ceiling of the PACIFIC trial? Ther. Adv. Med. Oncol. 14, 17588359221113268. 10.1177/17588359221113268 35923929 PMC9340398

[B159] Reutershan J. Cagnina R. E. Chang D. Linden J. Ley K. (2007). Therapeutic anti-inflammatory effects of myeloid cell adenosine receptor A2a stimulation in lipopolysaccharide-induced lung injury. J. Immunol. 179, 1254–1263. 10.4049/jimmunol.179.2.1254 17617618

[B160] Richter J. (1992). Effect of adenosine analogues and cAMP-raising agents on TNF-GM-CSF-and chemotactic peptide-induced degranulation in single adherent neutrophils. J. Leukoc. Biol. 51, 270–275. 10.1002/JLB.51.3.270 1371803

[B161] Rosen E. M. Fan S. Rockwell S. Goldberg I. D. Goldberg D. (1999). The molecular and cellular basis of radiosensitivity: implications for understanding how normal tissues and tumors respond to therapeutic radiation. Cancer Invest 17, 56–72. 10.1080/07357909909011718 10999050

[B162] Ruiz-Fernandez de Cordoba B. Martínez-Monge R. Lecanda F. (2023). ENPP1 immunobiology as a therapeutic target. Clin. Cancer Res. 29, 2184–2193. 10.1158/1078-0432.CCR-22-1681 36719675 PMC10261920

[B163] Ryzhov S. Novitskiy S. V. Goldstein A. E. Biktasova A. Blackburn M. R. Biaggioni I. (2011). Adenosinergic regulation of the expansion and immunosuppressive activity of CD11b+Gr1+ cells. J. Immunol. 187, 6120–6129. 10.4049/JIMMUNOL.1101225 22039302 PMC3221925

[B164] Saigí M. Mesía-Carbonell O. Barbie D. A. Guillamat-Prats R. (2023). Unraveling the intricacies of CD73/adenosine signaling: the pulmonary immune and stromal microenvironment in lung cancer. Cancers (Basel) 15, 5706. 10.3390/cancers15235706 38067409 PMC10705793

[B165] Salmi M. Jalkanen S. (2005). Cell-surface enzymes in control of leukocyte trafficking. Nat. Rev. Immunol. 5, 760–771. 10.1038/nri1705 16200079

[B166] Sankar K. Bryant A. K. Strohbehn G. W. Zhao L. Elliott D. Moghanaki D. (2022). Real world outcomes versus clinical trial results of durvalumab maintenance in veterans with stage III non-small cell lung cancer. Cancers (Basel) 14, 614. 10.3390/cancers14030614 35158881 PMC8833364

[B167] Sarkar O. S. Donninger H. Al Rayyan N. Chew L. C. Stamp B. Zhang X. (2023). Monocytic MDSCs exhibit superior immune suppression via adenosine and depletion of adenosine improves efficacy of immunotherapy. Sci. Adv. 9, eadg3736. 10.1126/sciadv.adg3736 37390211 PMC10313166

[B168] Sato M. Odagiri K. Tabuchi Y. Okamoto H. Shimokawa T. Nakamura Y. (2024). Patterns and incidence of pneumonitis and initial treatment outcomes with durvalumab consolidation therapy after radical chemoradiotherapy for stage III non-small cell lung cancer. Cancers (Basel) 16, 1162. 10.3390/cancers16061162 38539497 PMC10969599

[B169] Säve S. Mohlin C. Vumma R. Persson K. (2011). Activation of adenosine A2A receptors inhibits neutrophil transuroepithelial migration. Infect. Immun. 79, 3431–3437. 10.1128/IAI.05005-11 21646447 PMC3147561

[B170] Saze Z. Schuler P. J. Hong C. S. Cheng D. Jackson E. K. Whiteside T. L. (2013). Adenosine production by human B cells and B cell–mediated suppression of activated T cells. Blood 122, 9–18. 10.1182/BLOOD-2013-02-482406 23678003 PMC3701906

[B171] Schaue D. Kachikwu E. L. McBride W. H. (2012). Cytokines in radiobiological responses: a review. Radiat. Res. 178, 505–523. 10.1667/RR3031.1 23106210 PMC3723384

[B172] Schaue D. McBride W. H. (2012). T lymphocytes and normal tissue responses to radiation. Front. Oncol. 2, 119–128. 10.3389/fonc.2012.00119 23050243 PMC3445965

[B173] Schena F. Volpi S. Faliti C. E. Penco F. Santi S. Proietti M. (2013). Dependence of immunoglobulin class switch recombination in B cells on vesicular release of ATP and CD73 ectonucleotidase activity. Cell Rep. 3, 1824–1831. 10.1016/J.CELREP.2013.05.022 23770243

[B174] Schipler A. Iliakis G. (2013). DNA double-strand-break complexity levels and their possible contributions to the probability for error-prone processing and repair pathway choice. Nucleic Acids Res. 41, 7589–7605. 10.1093/nar/gkt556 23804754 PMC3763544

[B175] Schuler P. J. Saze Z. Hong C. S. Muller L. Gillespie D. G. Cheng D. (2014). Human CD4+CD39+ regulatory T cells produce adenosine upon co-expression of surface CD73 or contact with CD73+ exosomes or CD73+ cells. Clin. Exp. Immunol. 177, 531–543. 10.1111/cei.12354 24749746 PMC4226604

[B176] Serbina N. V. Salazar-Mather T. P. Biron C. A. Kuziel W. A. Pamer E. G. (2003). TNF/iNOS-producing dendritic cells mediate innate immune defense against bacterial infection. Immunity 19, 59–70. 10.1016/S1074-7613(03)00171-7 12871639

[B177] Shankar B. Naidoo J. (2018). PD-1 and PD-L1 inhibitor toxicities in non-small cell lung cancer. J. Thorac. Dis. 10, S4034-S4037. 10.21037/JTD.2018.09.46 30631548 PMC6297512

[B178] Shao L. Zhang Y. Shi W. Ma L. Xu T. Chang P. (2021). Mesenchymal stromal cells can repair radiation-induced pulmonary fibrosis via a DKK-1-mediated Wnt/β-catenin pathway. Cell Tissue Res. 384, 87–97. 10.1007/s00441-020-03325-3 33496879

[B179] Sheng Y. Chen K. Jiang W. Wu Z. Zhang W. Jing H. (2021). PD-1 restrains IL-17A production from γδ T cells to modulate acute radiation-induced lung injury. Transl. Lung Cancer Res. 10, 685–698. 10.21037/TLCR-20-838 33718014 PMC7947382

[B180] Shiravand Y. Khodadadi F. Kashani S. M. A. Hosseini-Fard S. R. Hosseini S. Sadeghirad H. (2022). Immune checkpoint inhibitors in cancer therapy. Curr. Oncol. 2022 29, 3044–3060. 10.3390/CURRONCOL29050247 PMC9139602 35621637

[B181] Silva-Cardoso S. C. Tao W. Fernández B. M. Boes M. Radstake T. R. D. J. Pandit A. (2020). CXCL4 suppresses tolerogenic immune signature of monocyte-derived dendritic cells. Eur. J. Immunol. 50, 1598–1601. 10.1002/EJI.201948341 32502279 PMC7586983

[B182] Silva-Vilches C. Ring S. Mahnke K. (2018). ATP and its metabolite adenosine as regulators of dendritic cell activity. Front. Immunol. 9, 2581. 10.3389/fimmu.2018.02581 30473700 PMC6237882

[B183] Sitkovsky M. V. Lukashev D. Apasov S. Kojima H. Koshiba M. Caldwell C. (2004). Physiological control of immune response and inflammatory tissue damage by hypoxia-inducible factors and adenosine A2A receptors. Annu. Rev. Immunol. 22, 657–682. 10.1146/ANNUREV.IMMUNOL.22.012703.104731 15032592

[B184] Slater L. Bartlett N. W. Haas J. J. Zhu J. Message S. D. Walton R. P. (2010). Co-ordinated role of TLR3, RIG-I and MDA5 in the innate response to rhinovirus in bronchial epithelium. PLoS Pathog. 6, e1001178. 10.1371/JOURNAL.PPAT.1001178 21079690 PMC2973831

[B185] Spigel D. R. Faivre-Finn C. Gray J. E. Vicente D. Planchard D. Paz-Ares L. (2022). Five-year survival outcomes from the PACIFIC trial: durvalumab after chemoradiotherapy in stage III non-small-cell lung cancer. J. Clin. Oncol. 40, 1301–1311. 10.1200/JCO.21.01308 35108059 PMC9015199

[B186] Stagg J. Golden E. Wennerberg E. Demaria S. (2023). The interplay between the DNA damage response and ectonucleotidases modulates tumor response to therapy. Sci. Immunol. 8, eabq3015. 10.1126/SCIIMMUNOL.ABQ3015 37418547 PMC10394739

[B187] Sugimoto T. Fujimoto D. Sato Y. Tamiya M. Yokoi T. Taniguchi Y. (2022). Prospective multicenter cohort study of durvalumab for patients with unresectable stage III non-small cell lung cancer and grade 1 radiation pneumonitis. Lung Cancer 171, 3–8. 10.1016/J.LUNGCAN.2022.07.005 35863254

[B188] Szabó C. Scott G. S. Virág L. Egnaczyk G. Salzman A. L. Shanley T. P. (1998). Suppression of macrophage inflammatory protein (MIP)-1alpha production and collagen-induced arthritis by adenosine receptor agonists. Br. J. Pharmacol. 125, 379–387. 10.1038/SJ.BJP.0702040 9786512 PMC1565610

[B189] Takedachi M. Qu D. Ebisuno Y. Oohara H. Joachims M. L. McGee S. T. (2008). CD73-Generated adenosine restricts lymphocyte migration into draining lymph nodes. J. Immunol. 180, 6288–6296. 10.4049/JIMMUNOL.180.9.6288 18424752 PMC2709497

[B190] Tan K. Zhu H. Zhang J. Ouyang W. Tang J. Zhang Y. (2019). CD73 expression on mesenchymal stem cells dictates the reparative properties via its anti-inflammatory activity. Stem Cells Int. 2019, 8717694. 10.1155/2019/8717694 31249602 PMC6525959

[B191] Taugner J. Käsmann L. Eze C. Rühle A. Tufman A. Reinmuth N. (2021). Real-world prospective analysis of treatment patterns in durvalumab maintenance after chemoradiotherapy in unresectable, locally advanced NSCLC patients. Invest. New Drugs 39, 1189–1196. 10.1007/s10637-021-01091-9 33704621 PMC8280025

[B192] Thiel M. Chouker A. (1995). Acting via A2 receptors, adenosine inhibits the production of tumor necrosis factor-alpha of endotoxin-stimulated human polymorphonuclear leukocytes. J. Lab. Clin. Med. 126, 275–282. 7665975

[B193] Thompson L. F. Eltzschig H. K. Ibla J. C. Van De Wiele C. J. Resta R. Morote-Garcia J. C. (2004). Crucial role for ecto-5′-nucleotidase (CD73) in vascular leakage during hypoxia. J. Exp. Med. 200, 1395–1405. 10.1084/JEM.20040915 15583013 PMC1237012

[B194] Tormo A. J. Gauchat J.-F. (2013). A novel role for STAT5 in DC: controlling the Th2-response. JAKSTAT 2, e25352. 10.4161/JKST.25352 24498539 PMC3906145

[B195] Trautmann A. (2009). Extracellular ATP in the immune system: more than just a “danger signal.”. Sci. Signal 2, pe6. 10.1126/SCISIGNAL.256PE6 19193605

[B196] Umapathy N. S. Fan Z. H. Zemskov E. A. Alieva I. B. Black S. M. Verin A. D. (2010). Molecular mechanisms involved in adenosine-induced endothelial cell barrier enhancement. Vasc. Pharmacol. 52, 199–206. 10.1016/J.VPH.2009.12.008 PMC3868371 20045081

[B197] Vafaei S. Zekiy A. O. Khanamir R. A. Zaman B. A. Ghayourvahdat A. Azimizonuzi H. (2022). Combination therapy with immune checkpoint inhibitors (ICIs); a new frontier. Cancer Cell Int. 22, 10.1186/s12935-021-02407-8 PMC8725311 34980128

[B198] Vanderheiden A. Ralfs P. Chirkova T. Upadhyay A. A. Zimmerman M. G. Bedoya S. (2020). Type I and type III interferons restrict SARS-CoV-2 infection of human airway epithelial cultures. J. Virol. 94, e00985-20. 10.1128/jvi.00985-20 32699094 PMC7495371

[B199] Vaupel P. Mayer A. (2016). Hypoxia-driven adenosine accumulation: a crucial microenvironmental factor promoting tumor progression. Adv. Exp. Med. Biol. 876, 177–183. 10.1007/978-1-4939-3023-4_22 26782210

[B200] Vaupel P. Multhoff G. (2016). Adenosine can thwart antitumor immune responses elicited by radiotherapy: therapeutic strategies alleviating protumor ADO activities. Strahlenther. Onkol. 192, 279–287. 10.1007/S00066-016-0948-1 26961686

[B201] Venkatesulu B. P. Mahadevan L. S. Aliru M. L. Yang X. Bodd M. H. Singh P. K. (2018). Radiation-induced endothelial vascular injury: a review of possible mechanisms. JACC Basic Transl. Sci. 3, 563–572. 10.1016/j.jacbts.2018.01.014 30175280 PMC6115704

[B202] Vigano S. Alatzoglou D. Irving M. Ménétrier-Caux C. Caux C. Romero P. (2019). Targeting adenosine in cancer immunotherapy to enhance T-cell function. Front. Immunol. 10, 925. 10.3389/FIMMU.2019.00925 31244820 PMC6562565

[B203] Volmer J. B. Thompson L. F. Blackburn M. R. (2006). Ecto-5′-Nucleotidase (CD73)-Mediated adenosine production is tissue protective in a model of bleomycin-induced lung injury. J. Immunol. 176, 4449–4458. 10.4049/JIMMUNOL.176.7.4449 16547283

[B204] Walker B. A. Rocchini C. Boone R. H. Ip S. Jacobson M. A. (1997). Adenosine A2a receptor activation delays apoptosis in human neutrophils. J. Immunol. 158, 2926–2931. 10.4049/jimmunol.158.6.2926 9058831

[B205] Walsh R. J. Sundar R. Lim J. S. J. (2023). Immune checkpoint inhibitor combinations—current and emerging strategies. Br. J. Cancer 128, 1415–1417. 10.1038/s41416-023-02181-6 36747017 PMC10070427

[B206] Wang J. Matosevic S. (2018). Adenosinergic signaling as a target for natural killer cell immunotherapy. J. Mol. Med. 2018 96, 903–913. 10.1007/S00109-018-1679-9 30069747

[B207] Wang L. Zhang J. Zhang W. Zheng M. Guo H. Pan X. (2024). The inhibitory effect of adenosine on tumor adaptive immunity and intervention strategies. Acta Pharm. Sin. B 14, 1951–1964. 10.1016/j.apsb.2023.12.004 38799637 PMC11119508

[B208] Wang Q. Nagarkar D. R. Bowman E. R. Schneider D. Gosangi B. Lei J. (2009). Role of double-stranded RNA pattern recognition receptors in rhinovirus-induced airway epithelial cell responses. J. Immunol. 183, 6989–6997. 10.4049/JIMMUNOL.0901386 19890046 PMC2920602

[B209] Wang R. Ma X. Zhang X. Jiang D. Mao H. Li Z. (2023). Autophagy-mediated NKG2D internalization impairs NK cell function and exacerbates radiation pneumonitis. Front. Immunol. 14, 1250920. 10.3389/fimmu.2023.1250920 38077388 PMC10704197

[B210] Wang Y. Xu G. Wang J. Li X. H. Sun P. Zhang W. (2017). Relationship of Th17/treg cells and radiation pneumonia in locally advanced esophageal carcinoma. Anticancer Res. 37, 4643–4647. 10.21873/anticanres.11866 28739765

[B211] Welihinda A. A. Kaur M. Greene K. Zhai Y. Amento E. P. (2016). The adenosine metabolite inosine is a functional agonist of the adenosine A2A receptor with a unique signaling bias. Cell Signal 28, 552–560. 10.1016/J.CELLSIG.2016.02.010 26903141 PMC4826793

[B212] Wennerberg E. Lhuillier C. Vanpouille-Box C. Pilones K. A. García-Martínez E. Rudqvist N. P. (2017). Barriers to radiation-induced *in situ* tumor vaccination. Front. Immunol. 8, 229. 10.3389/fimmu.2017.00229 28348554 PMC5346586

[B213] Wennerberg E. Spada S. Rudqvist N. P. Lhuillier C. Gruber S. Gruber S. (2020). CD73 blockade promotes dendritic cell infiltration of irradiated tumors and tumor rejection. Cancer Immunol. Res. 8, 465–478. 10.1158/2326-6066.CIR-19-0449 32047024 PMC7125001

[B214] Whitsett J. A. Alenghat T. (2014). Respiratory epithelial cells orchestrate pulmonary innate immunity. Nat. Immunol. 16, 27–35. 10.1038/ni.3045 PMC4318521 25521682

[B215] Wilson J. M. Ross W. G. Agbai O. N. Frazier R. Figler R. A. Rieger J. (2009). The A2B adenosine receptor impairs the maturation and immunogenicity of dendritic cells. J. Immunol. 182, 4616–4623. 10.4049/JIMMUNOL.0801279 19342636 PMC2989878

[B216] Wirsdörfer F. Cappuccini F. Niazman M. de Leve S. Westendorf A. M. Lüdemann L. (2014). Thorax irradiation triggers a local and systemic accumulation of immunosuppressive CD4+ FoxP3+ regulatory T cells. Radiat. Oncol. 9, 98–11. 10.1186/1748-717X-9-98 24766907 PMC4011772

[B217] Wirsdörfer F. De Leve S. Cappuccini F. Eldh T. Meyer A. V. Gau E. (2013). Extracellular adenosine production by ecto-5′-nucleotidase (CD73) enhances radiation-induced lung fibrosis. Cancer Res. 83, 3045–3056. 10.1158/0008-5472.CAN-15-2310 PMC4960984 26921334

[B218] Wirsdörfer F. De Leve S. Jendrossek V. (2019). Combining radiotherapy and immunotherapy in lung cancer: can we expect limitations due to altered normal tissue toxicity? Int. J. Mol. Sci. 20, 24–21. 10.3390/ijms20010024 PMC6337556 30577587

[B219] Wirsdörfer F. Jendrossek V. (2016). The role of lymphocytes in radiotherapy-induced adverse late effects in the lung. Front. Immunol. 7, 591. 10.3389/fimmu.2016.00591 28018357 PMC5155013

[B220] Wirsdörfer F. Jendrossek V. (2017). Modeling DNAdamage-induced pneumopathy in mice: insight from danger signaling cascades. Radiat. Oncol. 12, 142–225. 10.1186/s13014-017-0865-1 28836991 PMC5571607

[B221] Wunderlich R. Ruehle P. F. Deloch L. Unger K. Hess J. Zitzelsberger H. (2017). Interconnection between DNA damage, senescence, inflammation, and cancer. Front. Biosci. (Landmark Ed.) 22, 348–369. 10.2741/4488 27814618

[B222] Xia C. Yin S. To K. K. W. Fu L. (2023). CD39/CD73/A2AR pathway and cancer immunotherapy. Mol. Cancer 22, 44. 10.1186/s12943-023-01733-x 36859386 PMC9979453

[B223] Xu T. Wu L. Gandhi S. Jing W. Nguyen Q. N. Chen A. (2022). Treatment-related pulmonary adverse events induced by chemoradiation and Durvalumab affect survival in locally advanced non-small cell lung cancer. Radiotherapy Oncol. 176, 149–156. 10.1016/j.radonc.2022.10.002 PMC11813632 36209942

[B224] Yamaguchi T. Shimizu J. Oya Y. Watanabe N. Hasegawa T. Horio Y. (2022). Risk factors for pneumonitis in patients with non‐small cell lung cancer treated with immune checkpoint inhibitors plus chemotherapy: a retrospective analysis. Thorac. Cancer 13, 724–731. 10.1111/1759-7714.14308 35044093 PMC8888158

[B225] Yan Y. Fu J. Kowalchuk R. O. Wright C. M. Zhang R. Li X. (2022). Exploration of radiation-induced lung injury, from mechanism to treatment: a narrative review. Transl. Lung Cancer Res. 11, 307–322. 10.21037/tlcr-22-108 35280316 PMC8902083

[B226] Yang C. Liang Y. Liu N. Sun M. (2023). Role of the cGAS-STING pathway in radiotherapy for non-small cell lung cancer. Radiat. Oncol. 18, 145–147. 10.1186/s13014-023-02335-z 37667279 PMC10478265

[B227] Yang H. Yao F. Davis P. F. Tan S. T. Hall S. R. R. (2021). CD73, tumor plasticity and immune evasion in solid cancers. Cancers (Basel). 13, 177. 10.3390/cancers13020177 33430239 PMC7825701

[B228] Yang M. Ma C. Liu S. Shao Q. Gao W. Song B. (2010). HIF-dependent induction of adenosine receptor A2b skews human dendritic cells to a Th2-stimulating phenotype under hypoxia. Immunol. Cell Biol. 88, 165–171. 10.1038/icb.2009.77 19841638

[B229] Yap T. A. Parkes E. E. Peng W. Moyers J. T. Curran M. A. Tawbi H. A. (2021). Development of immunotherapy combination strategies in cancer. Cancer Discov. 11, 1368–1397. 10.1158/2159-8290.cd-20-1209 33811048 PMC8178168

[B230] Yarosz E. L. Chang C. H. (2018). The role of reactive oxygen species in regulating T cell-mediated immunity and disease. Immune Netw. 18, e14. 10.4110/IN.2018.18.E14 29503744 PMC5833121

[B231] Yegutkin G. G. (2008). Nucleotide- and nucleoside-converting ectoenzymes: important modulators of purinergic signalling cascade. Biochimica Biophysica Acta (BBA) - Mol. Cell Res. 1783, 673–694. 10.1016/J.BBAMCR.2008.01.024 18302942

[B232] Yegutkin G. G. Marttila-Ichihara F. Karikoski M. Niemelä J. Laurila J. P. Elima K. (2011). Altered purinergic signaling in CD73-deficient mice inhibits tumor progression. Eur. J. Immunol. 41, 1231–1241. 10.1002/EJI.201041292 21469131

[B233] Young A. Ngiow S. F. Barkauskas D. S. Sult E. Hay C. Blake S. J. (2016). Co-Inhibition of CD73 and A2AR adenosine signaling improves anti-tumor immune responses. Cancer Cell 30, 391–403. 10.1016/j.ccell.2016.06.025 27622332

[B234] Young A. Ngiow S. F. Gao Y. Patch A. M. Barkauskas D. S. Messaoudene M. (2018). A2AR adenosine signaling suppresses natural killer cell maturation in the tumor microenvironment. Cancer Res. 78, 1003–1016. 10.1158/0008-5472.CAN-17-2826 29229601

[B235] Zalavary S. Stendahl O. Bengtsson T. (1994). The role of cyclic AMP, calcium and filamentous actin in adenosine modulation of Fc receptor-mediated phagocytosis in human neutrophils. Biochim. Biophys. Acta 1222, 249–256. 10.1016/0167-4889(94)90176-7 8031862

[B236] Zanin R. F. Braganhol E. Bergamin L. S. Campesato L. F. I. Filho A. Z. Moreira J. C. F. (2012). Differential macrophage activation alters the expression profile of NTPDase and ecto-5′-nucleotidase. PLoS One 7, e31205. 10.1371/JOURNAL.PONE.0031205 22348056 PMC3278434

[B237] Zarek P. E. Huang C. T. Lutz E. R. Kowalski J. Horton M. R. Linden J. (2008). A2A receptor signaling promotes peripheral tolerance by inducing T-cell anergy and the generation of adaptive regulatory T cells. Blood 111, 251–259. 10.1182/BLOOD-2007-03-081646 17909080 PMC2200810

[B238] Zernecke A. Bidzhekov K. Özüyaman B. Fraemohs L. Liehn E. A. Lüscher-Firzlaff J. M. (2006). CD73/Ecto-5′-nucleotidase protects against vascular inflammation and neointima formation. Circulation 113, 2120–2127. 10.1161/CIRCULATIONAHA.105.595249 16636171

[B239] Zhan J. Huang L. Niu L. Lu W. Sun C. Liu S. (2024). Regulation of CD73 on NAD metabolism: unravelling the interplay between tumour immunity and tumour metabolism. Cell Commun. Signal. 22, 387. 10.1186/s12964-024-01755-y 39090604 PMC11292923

[B240] Zhou P. Chen L. Yan D. Huang C. Chen G. Wang Z. (2020). Early variations in lymphocytes and T lymphocyte subsets are associated with radiation pneumonitis in lung cancer patients and experimental mice received thoracic irradiation. Cancer Med. 9, 3437–3444. 10.1002/CAM4.2987 32207253 PMC7221303

[B241] Zhou S. Yang H. (2023). Immunotherapy resistance in non-small-cell lung cancer: from mechanism to clinical strategies. Front. Immunol. 14, 1129465. 10.3389/FIMMU.2023.1129465 37090727 PMC10115980

[B242] Zhou Y. Yan T. Zhou X. Cao P. Luo C. Zhou L. (2020). Acute severe radiation pneumonitis among non-small cell lung cancer (NSCLC) patients with moderate pulmonary dysfunction receiving definitive concurrent chemoradiotherapy: impact of pre-treatment pulmonary function parameters. Strahlenther. Onkol. 196, 505–514. 10.1007/s00066-019-01552-4 31828393

